# DeepLNM: A Multimodal Model for Predicting Lymph Node Metastasis in Colorectal Cancer

**DOI:** 10.1002/advs.77860

**Published:** 2026-10-14

**Authors:** Zhengyu Zhang, Chengyu Lu, Zhen Wang, Zhijian Cen, Yangshu Zhou, Danyi Li, Qi Xie, Bingbing Li, Shujing Guo, Zhijun Gan, Er‐ran Zhu, Jinbang Li, Aihetaimu Aimaier, Rui Mao, Weihao Qiu, Chenglong Zhao, Ying Tan, Xiaolin Liu, Huiting Ma, Tiansu Geng, Peiyue Wu, Huijun Du, Yu Wang, Yueping Liu, Li Liang

**Affiliations:** ^1^ Department of Pathology, Nanfang Hospital, School of Basic Medical Sciences Southern Medical University Guangzhou Guangdong China; ^2^ Guangdong Province Key Laboratory of Molecular Tumor Pathology Guangzhou Guangdong China; ^3^ Jinfeng Laboratory Chongqing China; ^4^ Department of Pathology, Zhujiang Hospital Southern Medical University Guangzhou Guangdong China; ^5^ Department of Radiology, The Affiliated Qingyuan Hospital (Qingyuan People's Hospital) Guangzhou Medical University Qingyuan Guangdong China; ^6^ Department of Pathology the Fourth Hospital of Hebei Medical University Shijiazhuang Hebei China

**Keywords:** colorectal cancer, deep learning, histopathology, lymph node metastasis, radiomics

## Abstract

Accurate assessment of lymph node metastasis (LNM) is critical for prognostic stratification and treatment selection in colorectal cancer (CRC), yet imaging and routine pathology can yield false‐negative/false‐positive results. We propose **DeepLNM**, a multi‐center foundation model for LNM prediction in CRC, evaluated with retrospective, external, prospective, and user study evidence. A multimodal dataset was assembled from three hospitals, with matched hematoxylin and eosin (H&E)‐stained whole‐slide images (WSI), CT scans, and text. DeepLNM achieved a mean AUC of 0.922 in the internal cohort and AUCs of 0.872 and 0.848 in two external cohorts. In a prospective cohort from a registered clinical trial (NCT07239063), DeepLNM remained robust (AUC = 0.845). In a user study, DeepLNM improved diagnostic accuracy, especially for junior pathologists (95.0%–97.3%), while reducing diagnostic time and increasing confidence. To explore the model's generalizability, it was further validated in a gastric cancer cohort, achieving an AUC of 0.793. Finally, DeepLNM identified histopathological features associated with LNM, addressing the “black‐box” concern of deep learning. Together, these results position DeepLNM as a clinically preliminarily validated multimodal model for predicting LNM status in CRC, potentially facilitating the clinical translation of AI pathology.

## Graphical abstract

Traditional diagnosis may miss positive LNM cases, and pathologists face a workload; therefore, we develop DeepLNM, a multimodal model that predicts lymph node metastasis in colorectal cancer from H&E WSIs, CT images, and text. DeepLNM is robust across internal, external, and prospective cohorts and, in a user study, improves diagnostic accuracy while reducing time and increasing confidence.

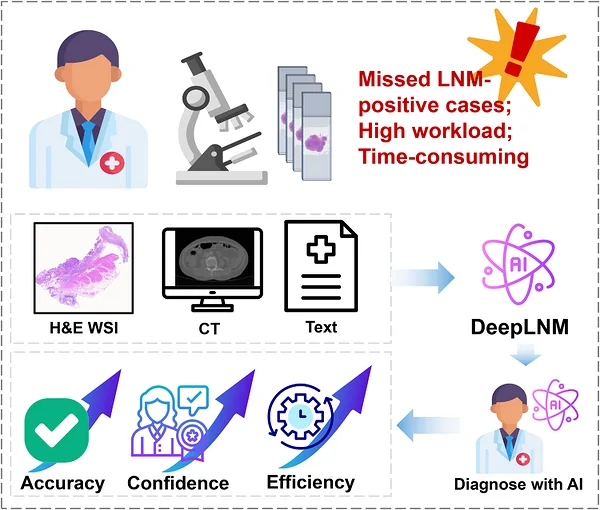

## 1 Introduction

Colorectal cancer (CRC) is one of the most formidable malignancies of the digestive system and the fourth leading cause of cancer‐related death worldwide, posing a major threat to human health [1]. The lymph node metastasis (LNM) status in CRC is pivotal to the clinical staging system, and the depth of primary tumor invasion (T stage) and regional LNM (N stage) are critical determinants in surgical planning [2]. The presence of LNM is strongly correlated with an increased risk of disease recurrence and cancer‐related mortality, thereby influencing the choice of individualized and precision treatment strategies for CRC patients [3, 4, 5].

Imaging examinations, such as computed tomography (CT) and magnetic resonance imaging (MRI), are currently the most commonly used approaches for preoperative assessment of LNM. Definitive determination of LNM status, however, relies largely on postoperative pathological examination, which is regarded as the “gold standard.” Notably, the number of lymph nodes retrieved intraoperatively may, with a certain probability, fail to meet the guideline‐recommended threshold of 12 [6, 7, 8]. More importantly, across healthcare systems worldwide—particularly in China—the growing volume of pathology specimens, coupled with a shortage of experienced pathologists, results in prolonged workloads and fatigue, which can compromise diagnostic performance and lead to missed micrometastases [9]. Junior pathologists may also struggle to distinguish tumor cells from histiocytes within lymph nodes, resulting in false‐positive assessments. Inaccurate pathological diagnosis, whether overly aggressive or overly conservative, may deprive patients of appropriate treatment options and substantially reduce postoperative 5‐year survival. Thus, the clinical imperative to refine the accuracy of LNM determination is clear. The development of a precise predictive system for LNM status is of paramount clinical importance, as it would significantly advance the prospects of individualized and precision treatment for CRC patients. This pursuit aligns with the overarching goal of improving patient outcomes and represents a critical step forward in the battle against CRC.

In recent years, artificial intelligence (AI) has demonstrated remarkable achievements in the medical field. Radiomics and pathomics, by extracting high‐throughput quantitative features from medical images, have established novel paradigms for decoding tumor phenotypes [10, 11, 12, 13, 14]. Current predictive models based on CT or whole slide imaging (WSI) have shown significant advantages in evaluating LNM in multiple cancer types [15, 16, 17, 18, 19]. Additionally, several pathology foundational models [20, 21, 22, 23] have incorporated LNM prediction tasks with commendable performance. However, existing research remains predominantly confined to unimodal analysis [24], failing to fully integrate microscopic pathological changes with macroscopic radiological phenotypic characteristics of tumors. Therefore, combining radiological and pathological images can facilitate the mining of latent features across modalities, enabling synergistic complementarity that may enhance predictions of tumor biological behavior.

In this study, we developed and validated DeepLNM, a multimodal model for predicting LNM status in patients with CRC. DeepLNM was trained on one private cohort and one public dataset, and externally validated in two independent cohorts. To further establish clinical utility in routine workflows, we conducted prospective validation in an independent prospective cohort and performed a user study demonstrating improvements in pathologists' diagnostic efficiency and workflow performance. To assess generalizability, we evaluated DeepLNM on previously unseen multimodal gastric cancer data, where the model also achieved promising performance. Collectively, these results support DeepLNM as a rigorously validated decision‐support tool with the potential to be integrated into routine pathology practice, enabling more accurate LNM assessment for pathologists and patients, as in Figure 1.

**FIGURE 1:**
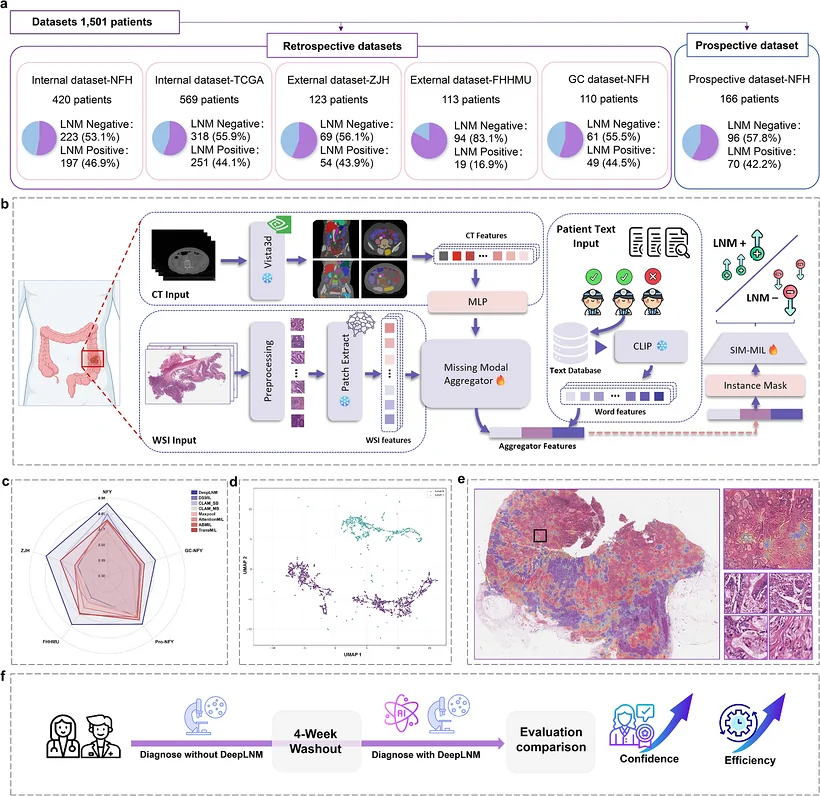
(a) The study analyzed a total of 1501 cases from retrospective and prospective datasets, including 1391 patients with colorectal cancer and 110 patients with gastric cancer. The retrospective dataset comprised internal training cohorts from NFH and TCGA, external validation cohorts from ZJH and FHHMU, and a gastric cancer cohort from NFH for exploratory validation. The prospective dataset was derived from NFH. (b) DeepLNM supports CT, WSI, and text inputs, and performs LNM prediction through feature extraction, cross‐modality fusion and prediction aggregation within the SIM‐MIL module. (c) DeepLNM ranked first in performance compared with seven other models across multiple cohorts. (d) UMAP visualization shows feature separation between LNM‐positive and LNM‐negative cases. (e) Visualization analyses indicate that DeepLNM attends to distinct histomorphological patterns across WSI classes. (f) DeepLNM supports pathologist‐assisted diagnosis: in a user study, pathologists reviewed the same set of lymph node WSIs under different diagnostic settings, with improvements in accuracy, efficiency and confidence. NFH, Nanfang Hospital, Southern Medical University; TCGA, The Cancer Genome Atlas; ZJH, Zhujiang Hospital, Southern Medical University; FHHMU, the Fourth Hospital of Hebei Medical University.

## 2 Results

### 2.1 Study Design and Patient Characteristics

In this study, we introduce DeepLNM, a multimodal model that predicts LNM status in CRC by integrating hematoxylin and eosin (H&E)‐stained whole‐slide image (WSI) of primary tumor, radiology images, and text‐based data. To support this task, we collected data from six cohorts comprising 1,501 patients (640 LNM positive cases, 42.6%; 861 LNM negative cases, 57.4%) for model training and validation.

With the exception of the public The Cancer Genome Atlas (TCGA) dataset, which includes WSI and text information only, all other cohorts provide paired WSI, CT, and text information, as shown in Figure 1a. We used the internal dataset‐NFH and TCGA for training and internal validation, including a total of 989 patients. Because TCGA lacks CT data, we performed missing‐data analyses for this component and shown in Figure 1b. Independent external cohorts included two retrospective cohorts: the ZJH cohort included 123 patients, and the FHHMU cohort included 113 patients. For generalizability evaluation, we collected multimodal gastric cancer data from NFH, comprising 110 patients, as well as a prospective cohort from the same institution that included 166 CRC patients. Notably, the prospective cohort was derived from a registered clinical trial at ClinicalTrials.gov (NCT07239063). All slides were digitized using a 40× scanner. CT examinations included contrast‐enhanced CT and non‐contrast CT, with imaging data retrievable in DICOM (DCM) format. Text information was obtained from patients' clinical medical records. Details of participant characteristics are presented in Table 1 and Table S1.

**TABLE 1:** Descriptive characteristics of patients with CRC (n=1391).

Characteristics	All (n=1391)	Cohorts					P Value	SMD
		Nanfang Hospital, Southern Medical University (n=420)	Zhujiang Hospital, Southern Medical University (n=123)	Fourth Hospital of Hebei Medical University (n=113)	TCGA (n=569)	Nanfang Hospital‐ Prospective Dataset (n=166)		
Age, Mean ± SD	63.127±12.732	61.090±12.136	59.911±13.827	61.416±10.192	66.202±12.792	61.289±12.655	<0.001	0.206
*Gender, n (%)*							<0.001	0.253
Female	630 (45.291)	159 (37.857)	72 (58.537)	65 (57.522)	272 (47.803)	62 (37.349)		
Male	761 (54.709)	261 (62.143)	51 (41.463)	48 (42.478)	297 (52.197)	104 (62.651)		
*Histological type, n (%)*							<0.001	0.381
Adenocarcinoma NOS	1274 (91.589)	388 (92.381)	113 (91.870)	113 (100.000)	496 (87.170)	164 (98.795)		
Mucinous Adenocarcinoma	91 (6.542)	11 (2.619)	7 (5.691)	0 (0.000)	73 (12.830)	0 (0.000)		
Mixed Adenocarcinoma	26 (1.869)	21 (5.000)	3 (2.439)	0 (0.000)	0 (0.000)	2 (1.205)		
*Histological grade, n (%)*							<0.001	2.433
G1	16 (1.150)	15 (3.571)	1 (0.813)	0 (0.000)	0 (0.000)	0 (0.000)		
G2	738 (53.055)	379 (90.238)	103 (83.740)	99 (87.611)	0 (0.000)	157 (94.578)		
G3	53 (3.810)	21 (5.000)	9 (7.317)	14 (12.389)	0 (0.000)	9 (5.422)		
MISS	584 (41.984)	5 (1.190)	10 (8.130)	0 (0.000)	569 (100.000)	0 (0.000)		
*Vessel carcinoma embolus, n (%)*							<0.001	0.430
YES	389 (27.965)	169 (40.238)	46 (37.398)	6 (5.310)	131 (23.023)	37 (22.289)		
NO	1002 (72.035)	251 (59.762)	77 (62.602)	107 (94.690)	438 (76.977)	129 (77.711)		
*Perineural invasion, n (%)*							<0.001	1.090
YES	448 (32.207)	211 (50.238)	78 (63.415)	17 (15.044)	57 (10.018)	85 (51.205)		
NO	595 (42.775)	209 (49.762)	45 (36.585)	96 (84.956)	164 (28.822)	81 (48.795)		
MISS	348 (25.018)	0 (0.000)	0 (0.000)	0 (0.000)	348 (61.160)	0 (0.000)		
*pT, n (%)*							<0.001	0.882
Tis	11 (0.791)	8 (1.905)	0 (0.000)	0 (0.000)	1 (0.176)	2 (1.205)		
T1	61 (4.385)	35 (8.333)	5 (4.065)	0 (0.000)	17 (2.988)	4 (2.410)		
T2	213 (15.313)	68 (16.190)	18 (14.634)	0 (0.000)	102 (17.926)	25 (15.060)		
T3	806 (57.944)	225 (53.571)	79 (64.228)	20 (17.699)	384 (67.487)	98 (59.036)		
T4	300 (21.567)	84 (20.000)	21 (17.073)	93 (82.301)	65 (11.424)	37 (22.289)		
*pN, n (%)*							<0.001	0.353
N0	800 (57.513)	223 (53.095)	69 (56.098)	94 (83.186)	318 (55.888)	96 (57.831)		
N1	358 (25.737)	137 (32.619)	27 (21.951)	12 (10.619)	137 (24.077)	45 (27.108)		
N2	233 (16.751)	60 (14.286)	27 (21.951)	7 (6.195)	114 (20.035)	25 (15.060)		
*Stage, n (%)*							<0.001	0.756
Stage 0	11 (0.791)	8 (1.905)	0 (0.000)	0 (0.000)	1 (0.176)	2 (1.205)		
Stage I	221 (15.888)	77 (18.333)	22 (17.886)	0 (0.000)	102 (17.926)	20 (12.048)		
Stage II	544 (39.109)	120 (28.571)	47 (38.211)	95 (84.071)	208 (36.555)	74 (44.578)		
Stage III	533 (38.318)	215 (51.190)	54 (43.902)	18 (15.929)	176 (30.931)	70 (42.169)		
Stage IV	82 (5.895)	0 (0.000)	0 (0.000)	0 (0.000)	82 (14.411)	0 (0.000)		
*CT, n (%)*							<0.001	0.844
Contrast‐enhanced CT	508 (36.520)	129 (30.714)	123 (100.000)	90 (79.646)	0 (0.000)	166 (100.000)		
Non‐contrast CT	314 (22.574)	291 (69.286)	0 (0.000)	23 (20.354)	0 (0.000)	0 (0.000)		
NA	569 (40.906)	0 (0.000)	0 (0.000)	0 (0.000)	569 (100.000)	0 (0.000)		

### 2.2 A Novel Framework for LNM Prediction

The DeepLNM framework integrates pathological, radiological, and clinical insights through three phases: feature extraction, cross‐modal fusion, and predictive aggregation, as in Figure 2 and Figures S1–S3. First, specialized pre‐trained encoders—inspired by state‐of‐the‐art vision‐language architectures [25, 26]—extract high‐dimensional embeddings from WSIs, 3D CT scans, and clinical records. We utilized a Missing Modality Aggregator to ensure robustness [27, 28]. Finally, a Stochastic Instance Masking Multiple Instance Learning (SIM‐MIL) module, building upon the attention‐based Multiple Instance Learning (MIL) framework [29], aggregates these features into a patient‐level probability score.

**FIGURE 2:**
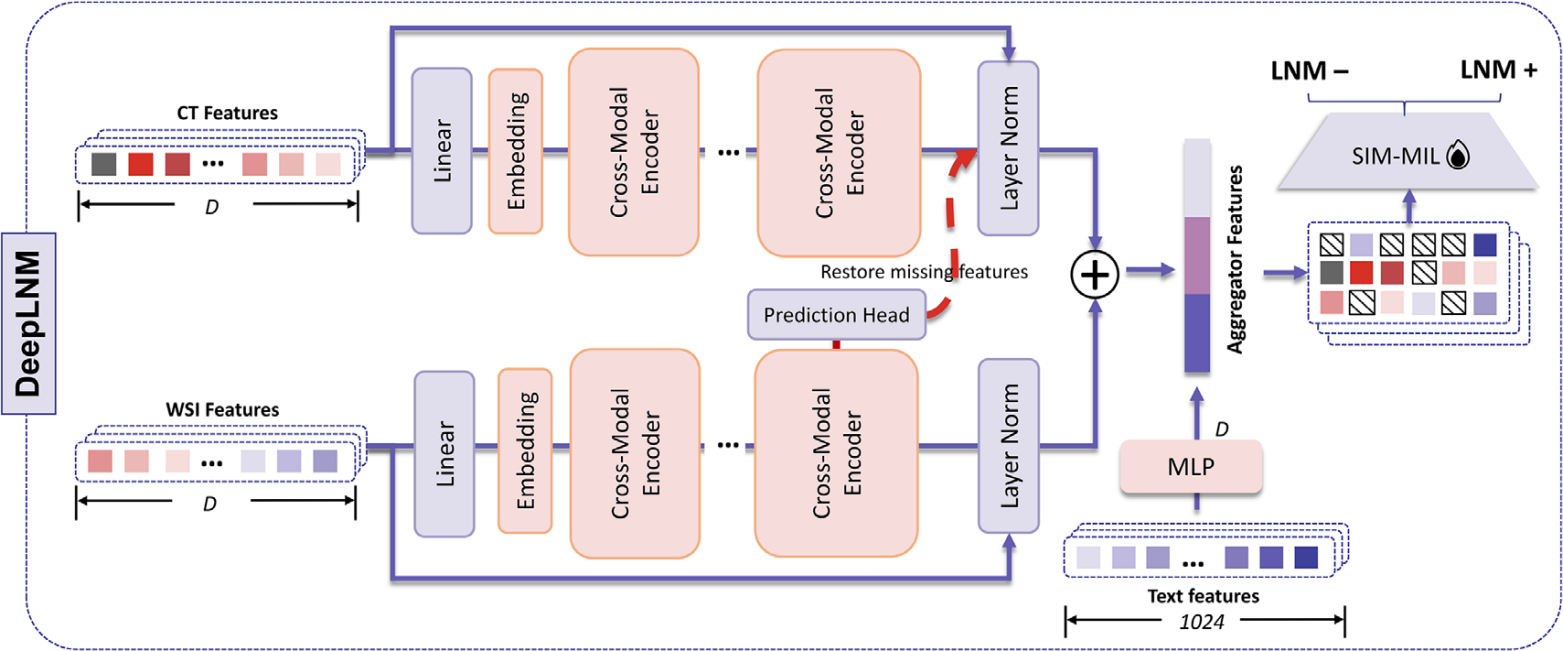
The framework employs a multi‐modal fusion strategy integrating CT imaging features, WSI pathological features, and clinical text features. The CT and WSI features are processed through parallel streams comprising linear projection, embedding layers, and stacked Cross‐Modal Encoders to capture inter‐modal interactions. Specifically, a feature restoration mechanism equipped with a Prediction Head is incorporated into the WSI branch to effectively mitigate the issue of missing modality data. The visual representations, normalized via Layer Norm, are fused through element‐wise addition and subsequently aggregated with clinical text features encoded by a multi‐layer perceptron. Finally, the aggregated masked instance features are input into the Stochastic Instance Masking Multiple Instance Learning (SIM‐MIL) module to predict Lymph Node Metastasis (LNM) status (LNM− vs. LNM+).

### 2.3 The Performance of DeepLNM

Our work aims to reduce the workload of pathologists and radiologists; therefore, we adopted a weakly supervised annotation strategy for model training and evaluation (Section 5). The most critical feature of DeepLNM is its ability to predict LNM status by extracting morphological characteristics from CT scans, text, and WSIs. We used multimodal data from the private NFH cohort and the public TCGA dataset as internal data for model training. To increase sample size, although TCGA contains only WSI and text information, we appropriately handled this limitation using a missing‐data strategy. Using fivefold cross‐validation, DeepLNM achieved a mean area under the curve (AUC) of 0.922 and an accuracy of 0.871, as in Figure 3a,b. We compared DeepLNM with five widely used pathology foundation models in the internal cohort. DeepLNM again ranked first, slightly higher than the next best model, Prov‐Gigapath (AUC:0.915), detailed results are provided in Table S2. To further evaluate applicability across institutions, we performed external validation in two independent cohorts, ZJH and FHHMU, achieving AUCs of 0.872 and 0.848, respectively, as shown in Figure 3e,f, supporting model robustness across datasets.

**FIGURE 3:**
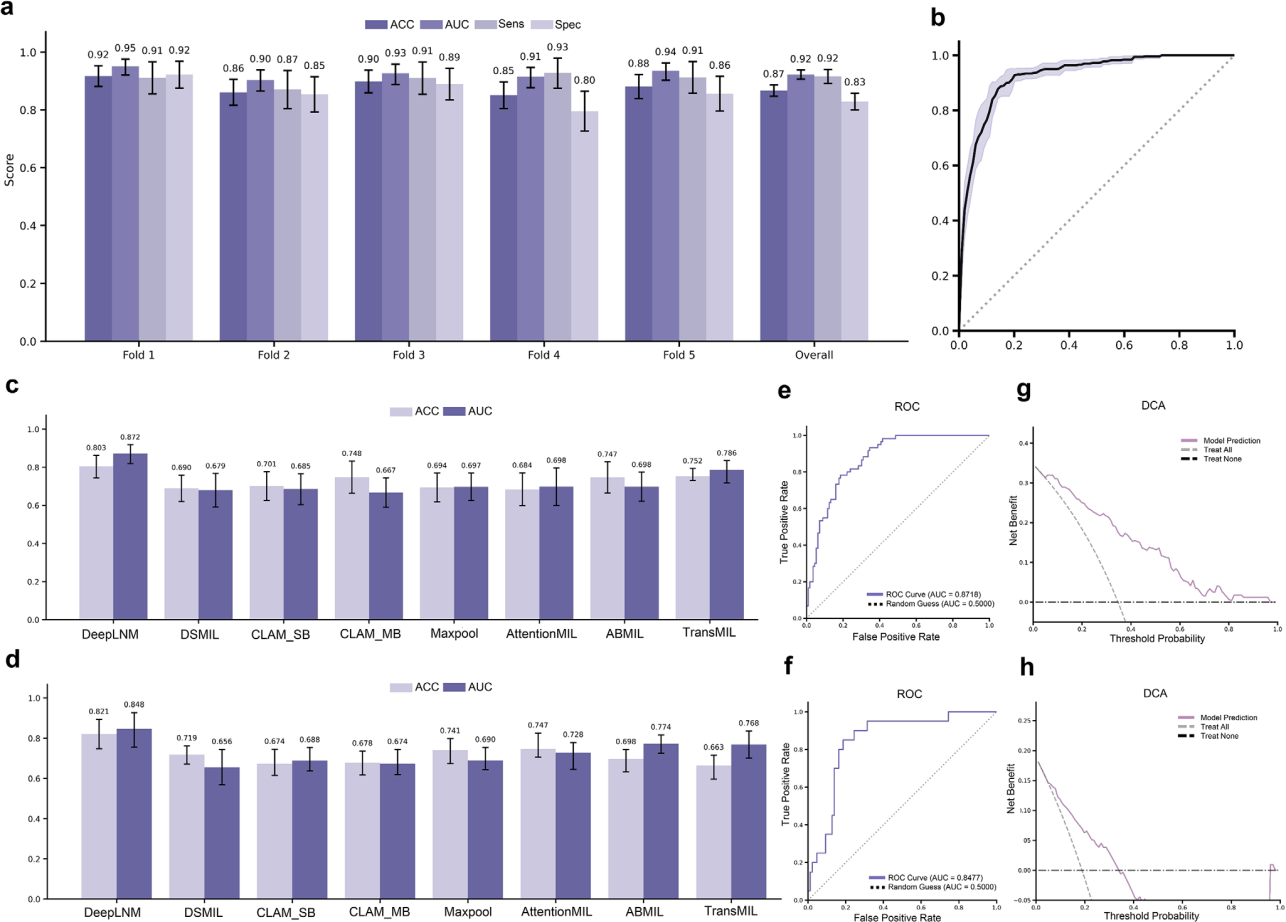
(a) Fivefold cross‐validation results on the internal dataset. (b) AUC on the internal dataset. (c) Benchmarking results on the external ZJH cohort. (d) Benchmarking results in the external FHHMU cohort. (e) AUC on the external ZJH cohort. (f) AUC on the external FHHMU cohort. (g) DCA on the external ZJH cohort. (h) DCA on the external FHHMU cohort. ZJH, Zhujiang Hospital, Southern Medical University; FHHMU, the Fourth Hospital of Hebei Medical University.

MIL is among the most widely used frameworks in computational pathology, covering tasks such as subtype analysis, diagnosis, and prognostic prediction, and several MIL models have reported performance in LNM prediction. To benchmark DeepLNM against existing approaches, we selected seven mainstream attention‐based MIL models and evaluated them in the two independent external validation cohorts, as shown in Figure 3c,d and Tables S3 and S4. In the ZJH cohort, DeepLNM achieved the highest AUC, which was 0.872, exceeding the second‐ranked TransMIL (AUC:0.786), whereas the AUCs of the other six models ranged from 0.667 to 0.698; these differences were statistically significant. Similar results were observed in the FHHMU cohort.

Next, we compared the multimodal model with unimodal models. As shown in Table S5, the multimodal model integrating CT, WSI, and text achieved higher AUC (0.928), sensitivity (0.892), and specificity (0.918) than unimodal models. Specifically, it outperformed the text‐only model (AUC: 0.691), WSI‐only model (AUC: 0.895), and CT‐only model (AUC: 0.835). These results demonstrate that DeepLNM can effectively fuse macroscopic radiological features with microscopic pathological features to enable accurate prediction of LNM status. Collectively, these comparisons across multiple cohorts and models indicate that DeepLNM provides a notable advance for predicting LNM status in CRC.

To assess clinical utility beyond discriminative accuracy, we performed decision curve analysis (DCA) for LNM‐negative and LNM‐positive classification. DCA showed a consistent positive net benefit across all probability thresholds in both external cohorts, compared with the treat‐all and treat‐none strategies in Figure 3g,h, indicating that DeepLNM‐assisted decision‐making can improve clinical diagnostic outcomes across institutions.

### 2.4 The Generalization Performance of the Model

A pathology AI model intended for clinical translation must maintain strong performance on previously unseen data. To evaluate the generalizability of DeepLNM, we collected prospective multimodal CRC data from a clinically registered study (NCT07239063). Although several radiology‐based models have been prospectively validated, to our knowledge, DeepLNM is the first model to prospectively validate LNM prediction in CRC using multimodal data that include CT, WSI, and text. Our prospective multimodal dataset was derived from patients who underwent surgical resection for CRC at NFH between September 2025 and December 2025, ensuring complete independence from earlier model training and validation. In this prospective cohort, DeepLNM achieved an AUC of 0.845, as shown in Figure 4a. We performed DCA on the prospective cohort. The results showed that, across threshold probabilities from 12% to 98%, the decision curve remained above the treat‐all and treat‐none strategies, indicating potential clinical utility, as shown in Figure 4b. In addition, a post hoc exploratory analysis of patients with initially insufficient lymph node retrieval in the prospective cohort was performed, and the detailed results are provided in the Supporting Information.

**FIGURE 4:**
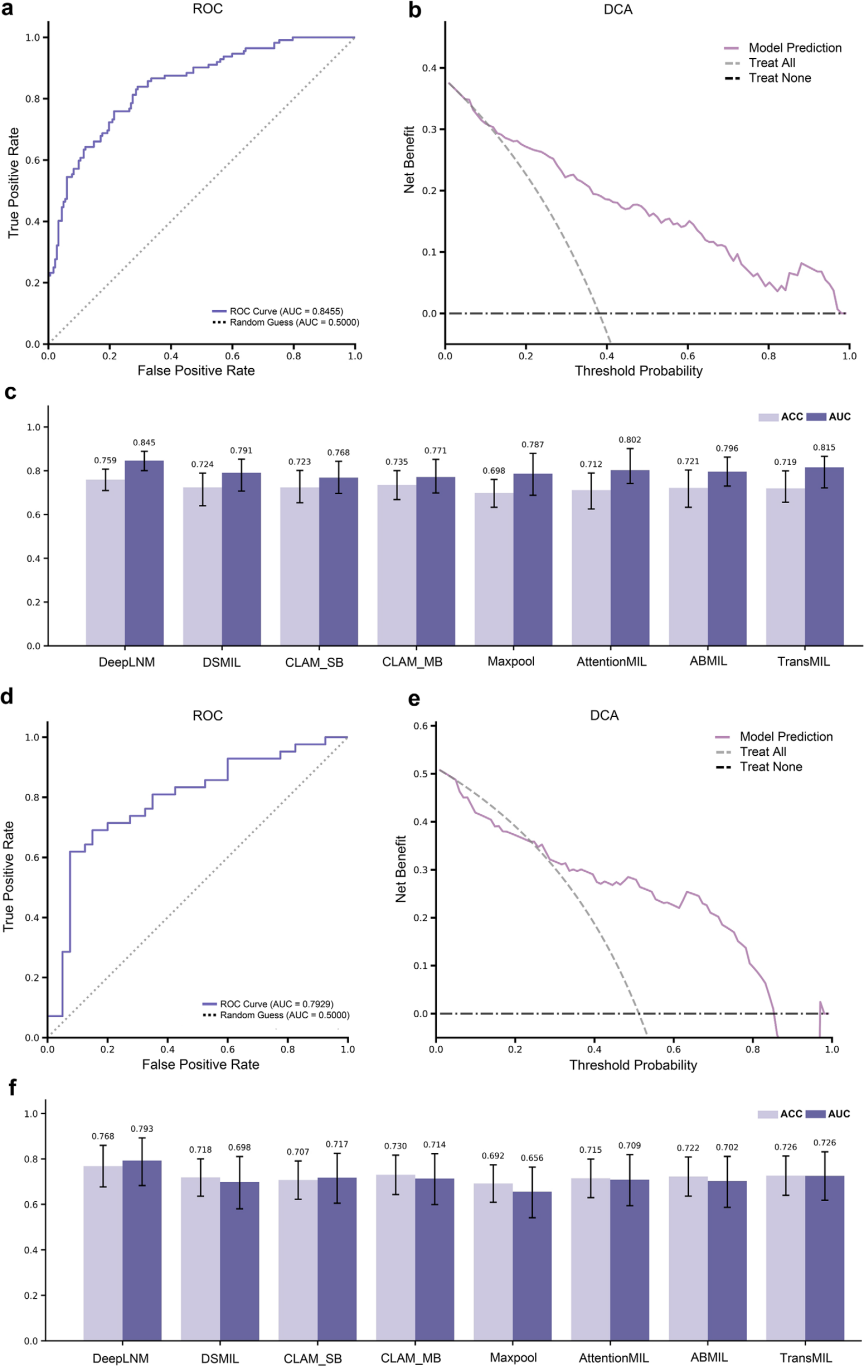
(a) AUC on the prospective cohort. (b) DCA on the prospective cohort. (c) Benchmarking results on the prospective cohort. (d) AUC on the gastric cancer cohort. (e) DCA on the gastric cancer cohort. (f) Benchmarking results on the gastric cancer cohort.

Next, we benchmarked DeepLNM against the seven mainstream attention‐based MIL models described above. DeepLNM remained ranked first among all models, further supporting its strong generalizability and feasibility in Figure 4c and Table S6.

To assess the impact of the number of WSIs per patient, we randomly selected 53 patients from the prospective cohort and additionally scanned all tumor tissue slides, including 30 LNM‐negative patients (123 WSIs) and 23 LNM‐positive patients (96 WSIs). Performance was compared between single‐WSI and multi‐WSIs settings. Bootstrap resampling revealed no statistically significant differences in sensitivity, specificity, accuracy, or AUC between the two settings (*p* > 0.05; Table S7).

In routine pathology practice for digestive system tumors, pathologists diagnose LNM not only in CRC but also in gastric cancer specimens. However, gastric cancer exhibits pronounced histological specificity, and poorly differentiated adenocarcinomas such as mucinous adenocarcinoma and signet‐ring cell carcinoma are common. During lymph node assessment, tumor cells from some poorly differentiated adenocarcinomas may be distributed within the lymph node capsule and can closely resemble normal tissue cells and adipocytes, making discrimination challenging. As a result, diagnosing LNM status in gastric cancer is time‐consuming and prone to missed diagnoses or misclassification. Although DeepLNM was trained and validated on CRC data, we collected multimodal gastric cancer data from NFH to test its applicability across cancer types and to help reduce the burden of LNM assessment in gastric cancer. DeepLNM achieved an AUC of 0.793, in Figure 4d. DCA indicated that, across threshold probabilities from 22% to 86%, the decision curve was above the treat‐all and treat‐none strategies, as shown in Figure 4e. Although performance decreased slightly, comparison with the other seven models showed that DeepLNM still outperformed all alternatives in Figure 4f and Table S8. These findings suggest that DeepLNM can predict LNM status in CRC and shows potential for application to gastric cancer, supporting its cross‐cancer generalizability.

### 2.5 User Study: DeepLNM Improves Diagnostic Confidence, Accuracy, and Efficiency

To accelerate the clinical translation of DeepLNM, we designed a rigorous user study to evaluate its performance in real‐world diagnostic settings. We randomly selected 136 patients from the NFH‐PRO cohort and collected all lymph node WSIs for these cases. Details of participant characteristics are presented in Tables S9 and S10.

As shown in Figure 5a–c and Table S11, with DeepLNM assistance, the diagnostic accuracy of six pathologists increased (one senior pathologist achieved 100% accuracy in both rounds). Among junior pathologists, diagnostic accuracy improved significantly from 95.0% to 97.3%. In addition, diagnostic efficiency improved markedly for all pathologists, particularly junior pathologists, whose mean reading time decreased from 152.2 to 120.3 s, as shown in Figure 5b. In terms of diagnostic confidence, DeepLNM substantially increased confidence among junior pathologists, with the mean score rising from 8.5 to 9.2, in Figure 5c.

**FIGURE 5:**
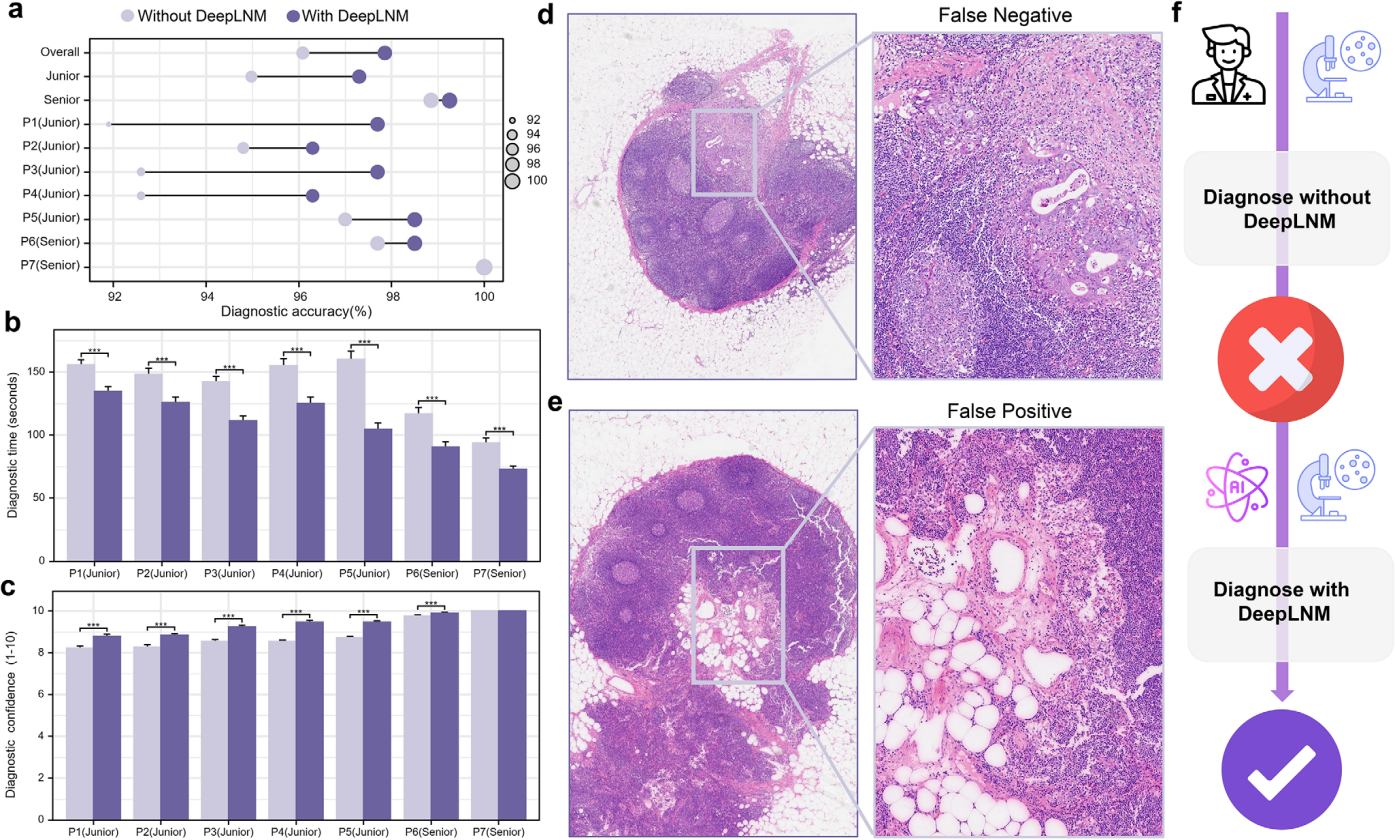
(a) Accuracy improvements for seven pathologists with different levels of experience with DeepLNM assistance. (b) Comparison of diagnostic time with and without DeepLNM assistance across the seven pathologists. (c) Changes in diagnostic confidence with DeepLNM assistance. (d–f) Representative cases showing that, with model assistance, pathologists re‐evaluated previously misclassified cases more accurately, reducing false‐negative and false‐positive assessments.

We further analyzed cases that were correctly classified with model assistance. We found that junior pathologists frequently overlooked tumor foci that were morphologically similar to germinal centers, leading to false‐negative assessments, as shown in Figure 5d. In some cases, less experienced pathologists misinterpreted fibrous connective tissue and distorted histiocytes as tumor cells, resulting in false‐positive assessments, as shown in Figure 5e. Collectively, these findings indicate that DeepLNM can support pathologists across multiple dimensions in making correct diagnoses, particularly for junior pathologists, by reducing the burden of tedious and complex LNM assessment (Figure 5f), thereby demonstrating promising potential for clinical deployment.

### 2.6 Visualizing Deep Learning‐Based Predictions

Finally, we employed visualization techniques to interpret how the model makes predictions, a crucial step for clinical adoption of deep learning in pathology [30, 31]. In the internal validation set, we used the Integrated Gradients method [32] to evaluate the relative contributions of all pathological patches to the DeepLNM prediction results. The IG scores were converted into heatmaps, enabling intuitive identification of pathological features associated with LNM status. In the predicted LNM negative group, the top 50 patches with the highest IG scores predominantly exhibited features such as lymphocytic infiltration, well‐differentiated glandular structures, and adipocyte infiltration—characteristics associated with lower tumor aggressiveness and favorable clinical outcomes, as shown in Figure 6a. Conversely, in the predicted LNM‐positive group, the top 50 IG‐scored patches displayed aggressive tumor features, including poorly differentiated tumor cells, desmoplastic reaction, and necrosis in Figure 6b. To quantitatively analyze the above observations, we additionally randomly selected 60 correctly predicted patients from the internal validation set, including 30 LNM‐positive and 30 LNM‐negative patients. For each patient, the 10 patches with the highest IG scores were selected, yielding 600 patches for histomorphological review by two senior pathologists. The distribution of histological features differed significantly between the LNM‐positive and LNM‐negative groups (Table S12). Poorly differentiated glandular structures were most prevalent in high‐IG patches from LNM‐positive patients (43.3%), whereas lymphocytic infiltration was the predominant feature in those from LNM‐negative patients (26.3%). These results quantitatively support that DeepLNM highlights distinct histomorphological patterns associated with LNM status.

**FIGURE 6:**
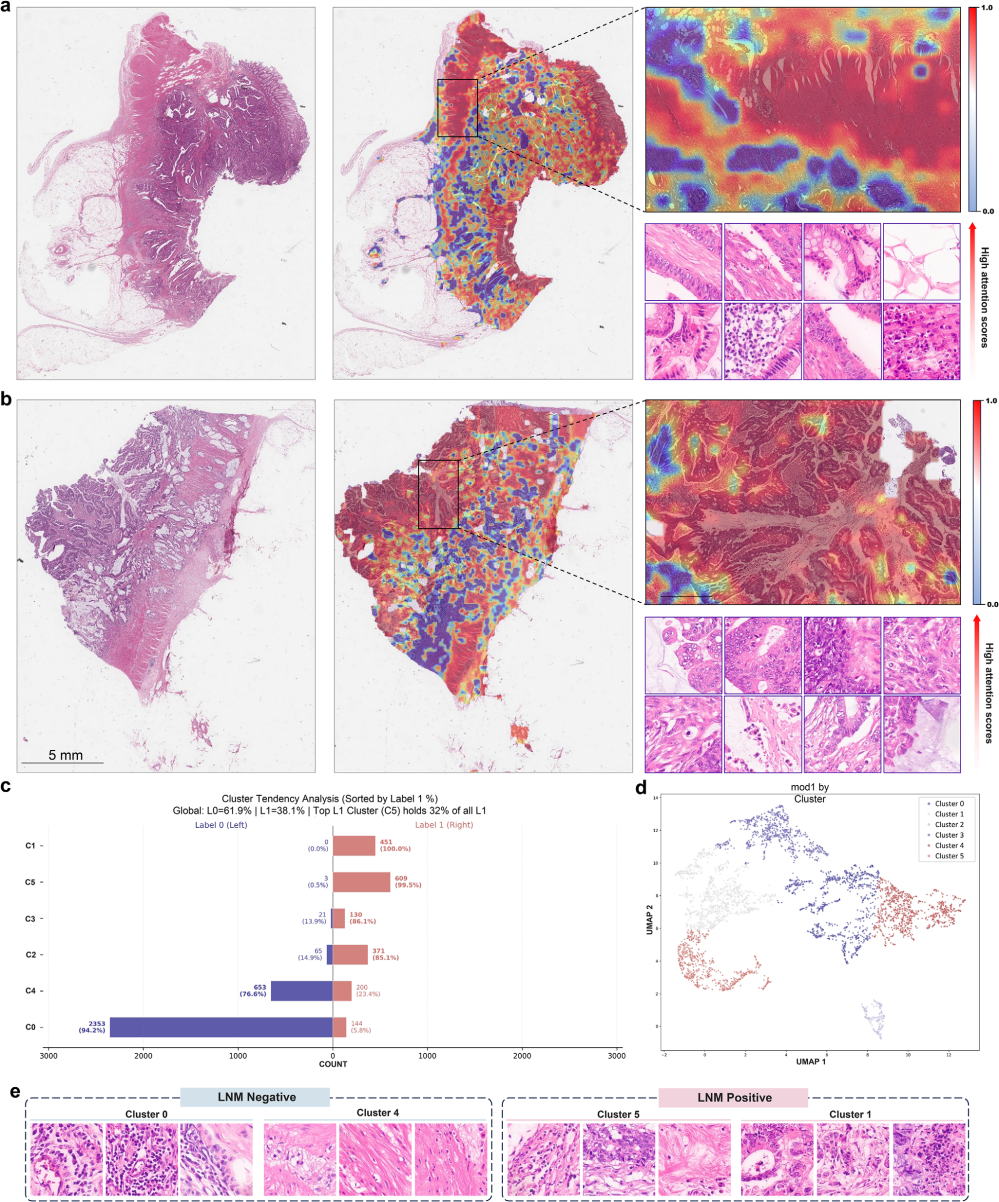
Attention heatmaps for correctly predicted LNM‐negative (a) and LNM‐positive (b) cases in the internal dataset. DeepLNM computed attention scores for all patches, with higher scores indicating greater attention. Heatmaps were overlaid on WSIs, and representative regions were magnified to highlight histopathological features with high attention (red) and low attention (blue). (c–e) Dimensionality‐reduction and clustering analyses: DeepLNM grouped patches into five distinct histomorphological subgroups according to LNM status. In LNM‐negative WSIs, the model focused more on C0 and C4, corresponding to lymphocyte infiltration and normal neuromuscular tissue (e, left). In LNM‐positive WSIs, the model focused more on C5 and C1, corresponding to poorly differentiated tumor cells and necrotic nuclear debris (e, right).

Complementing the previous integrated gradients (IG) analysis, we further employed unsupervised clustering techniques to interpret the high‐dimensional feature representations learned by the model, aligning with established paradigms in computational pathology foundation models [21, 33]. The cluster tendency analysis (Figure 6c) revealed a marked stratification of patches based on clinical LNM status. Clusters 1 and 5 were highly specific for the LNM positive group (Label 1), with Cluster 1 composed entirely of positive patches (100%) and Cluster 5 reaching 99.5% positivity. Conversely, Clusters 0 and 4 were predominantly enriched in the LNM‐negative group (Label 0). This distinct separation based on labels was visually corroborated by the UMAP projection (Figure 6d), where the clusters formed well‐defined, isolated territories within the feature space, reflecting the model's ability to encode discriminative histological signatures [22, 23].

We subsequently examined the histological morphology of these representative clusters to validate their biological relevance in Figure 6e. Patches within the LNM negative associated clusters (Clusters 0 and 4) predominantly exhibited non‐neoplastic characteristics indicating lower aggressiveness. For instance, Cluster 4 largely displayed abundant stromal connective tissue, while Cluster 0 showed benign cellular elements lacking significant atypia. In strong contrast, the LNM positive associated clusters (Clusters 5 and 1) captured aggressive tumor features. Cluster 5 frequently presented distinct tumor nests, whereas Cluster 1 was characterized by poorly differentiated tumor cells with disorganized architecture. These findings indicate that the model successfully distinguishes between benign stromal or non‐neoplastic features and high‐risk metastatic morphologies to determine LNM status, consistent with recent advances in interpretable multimodal prognostic models [12, 13].

## 3 Discussion

Studies have shown that poor prognosis in patients with CRC is associated with LNM positivity and that the 5‐year overall survival of LNM‐positive patients is significantly lower than that of LNM‐negative patients. Therefore, accurate diagnosis of LNM status is critical for selecting individualized treatment strategies. Preoperative imaging examinations are crucial for assessing LNM status. CT can infer nodal metastasis based on lymph node location and size, morphological regularity, and enhancement characteristics. However, small metastatic foci may be easily overlooked, leading to misdiagnosis. Conventional LNM assessment largely relies on pathologists manually reviewing surgically resected lymph nodes under a microscope. Because expertise varies across pathologists, particularly among junior pathologists with limited experience, diagnostic accuracy and reliability are not always satisfactory. Even experienced senior pathologists are often overloaded; prolonged lymph node examination under the microscope can lead to missed metastatic tumor cells owing to visual fatigue, small metastatic foci, and other factors, resulting in missed positive LNM findings and staging bias. In addition, a sufficient number of lymph nodes may not always be retrieved during pathological specimen sampling, resulting in fewer than 12 examined lymph nodes and potentially increasing the risk of inaccurate N staging. Accordingly, there is an urgent need for AI‐assisted prediction of LNM status in CRC, and DeepLNM largely addresses this need.

DeepLNM demonstrates excellent performance in predicting LNM status in CRC and shows strong generalizability in independent external cohorts and a prospective cohort. Our results indicate that DeepLNM has the potential to become a practical tool for broad clinical use. Because it is often difficult to obtain multiple modalities from the same patient simultaneously, cohorts used in existing studies are typically single‐source, small in scale, and limited in generalizability and interpretability. In this study, we collected retrospective multimodal data from CRC patients, including text information, CT scans, and WSIs, from three private institutions spanning northern and southern China, as well as from one public database. We also included multimodal gastric cancer data and prospective multimodal CRC data from the same private institution. By integrating retrospective data with real‐world prospective data from a clinically registered study (NCT07239063), we ensured the diversity and practical relevance of our large‐scale dataset, which is essential for developing AI models that can be deployed across diverse clinical settings while maintaining robust performance. This is the first multimodal, multicenter AI model for predicting CRC LNM status that incorporates a prospective cohort and is evaluated in real‐world workflow settings.

DeepLNM also introduces methodological advancements. Due to the scarcity of public datasets providing multimodal data, especially CT information, we designed a two‐stage fusion module to integrate multimodal inputs. We addressed the issue of missing CT modality through mask prediction [34], thereby enhancing cross‐modal context. We solved the prediction of LNM using SIM‐MIL. DeepLNM achieved satisfactory results. It reached an AUC of 0.922 in the internal test set and achieved AUCs of 0.872 and 0.848 in two independent external datasets. Although performance declined slightly in external cohorts, benchmarking against seven mainstream MIL models showed that DeepLNM ranked first, indicating strong generalizability. Decision curve analysis showed a consistent positive net benefit across all probability thresholds, demonstrating that DeepLNM‐assisted decision‐making can appropriately improve hospital diagnostic outcomes and highlighting its clinical utility.

Although multiple prior models can predict LNM status effectively [24], they have all been trained and validated on retrospective data. To demonstrate that the model can maintain strong performance in real clinical use when encountering previously unseen data, we prospectively collected multimodal CRC data from NFH; DeepLNM achieved the best performance in this cohort and in comparisons with other models. Clinically, LNM diagnosis in gastric cancer faces challenges similar to those in CRC. Accordingly, we evaluated DeepLNM in an additional multimodal gastric cancer cohort, where it achieved an AUC of 0.793 and again ranked first among the compared models. Nevertheless, DeepLNM showed reduced performance in the gastric cancer cohort compared with the CRC cohorts. This decrease may be related to differences between CRC and gastric cancer in pathological histomorphology, tissue architecture, tumorigenesis and progression, and the molecular mechanisms underlying LNM. Predicting LNM status in gastric cancer remains a considerable challenge. Future studies will incorporate data from multiple cancer types and apply model fine‐tuning and iterative optimization to improve the generalizability of DeepLNM across different cancers.

In recent years, an increasing number of pathology foundation models, such as CONCH [21], UNI [33], TITAN [22], and GPFM [23], have shown strong performance in pan‐cancer diagnostic and prediction tasks using approaches such as self‐supervised learning [35]. However, because they are not trained with multimodal data, their performance can be slightly inferior on vertical tasks within a single disease. We evaluated five pathology foundation models on two external datasets, and notably, DeepLNM still achieved the top performance. Future development of pathology models should further leverage foundation models for deeper analysis of multimodal vertical tasks [12, 20].

In terms of clinical application, a user study involving seven pathologists demonstrated the potential of DeepLNM to assist diagnostic workflows in real‐world clinical settings. DeepLNM may help address challenges associated with the uneven distribution of medical resources and shortages of pathologists in China by improving the efficiency and accuracy of lymph node assessment and increasing diagnostic confidence. These advantages make DeepLNM particularly valuable for resource‐limited primary hospitals, where experienced pathologists are scarce and delivering high‐quality care is challenging. DeepLNM is also applicable to high‐volume hospitals by providing predictive results that reduce diagnostic burden [30, 31]. Notably, DeepLNM is intended as a decision‐support tool rather than a replacement for pathologists; the final diagnosis would still be jointly determined by junior and senior pathologists, and pathology reports would be issued according to the standard sign‐out procedure.

We also performed a post hoc exploratory analysis to evaluate the potential utility of DeepLNM in patients with initially insufficient lymph node retrieval. Although all patients had at least 12 examined lymph nodes when the final pathology reports were issued, 24 patients in the prospective cohort had fewer than 12 lymph nodes retrieved during the initial sampling and therefore underwent additional sampling. In this subgroup, DeepLNM showed encouraging performance for final LNM status after completion of additional sampling. Notably, among patients who were initially assessed as LNM‐negative despite insufficient lymph node retrieval, DeepLNM correctly identified all patients who were subsequently found to have LNM after additional sampling. These findings suggest that DeepLNM may provide complementary risk information when initial lymph node retrieval is insufficient and may help alert pathologists to cases that require more careful lymph node retrieval or additional sampling.

Our study also mitigates the common “black‐box” limitation of deep learning. By generating visual heatmaps using Integrated Gradients and performing UMAP‐based clustering, we conducted histomorphological analyses of different LNM classifications (Figure 6, Figure S4, and Table S12). In LNM positive WSIs, the model focused more on necrotic tissue, desmoplastic reactions and poorly differentiated glands, whereas in LNM negative cases it focused more on well‐differentiated glands and infiltrating lymphocytes, consistent with prior findings. Additionally, we presented tumor WSIs, corresponding heatmaps, and representative lymph node WSIs for cases with false‐positive and false‐negative predictions by the model (Figure S5).

Despite these encouraging results, this study has limitations. First, although prospective clinical validation was included, the number of prospective cohorts remains limited, and more compelling clinical evidence is needed. Second, the private cohorts were all derived from China, and broader generalizability across other populations remains to be established; private cohorts from additional countries are needed to further improve generalizability. Looking ahead, incorporating more real‐world data and conducting more rigorous randomized controlled trials, such as randomized crossover trials, may further strengthen the level of evidence supporting DeepLNM and accelerate its implementation in routine clinical workflows. Future work should also further optimize the model architecture, for example by adopting lighter‐weight backbones, to facilitate the development and clinical deployment of more portable and efficient tools.

## 4 Methods

### 4.1 Study Participants

To train and validate the model, we collected multimodal data from 1501 patients across 6 cohorts. The training set included a retrospective internal cohort from Nanfang Hospital (NFH), Southern Medical University (*n* = 420; January 2022 to December 2024), and a public cohort from TCGA (*n* = 569). Two independent external validation cohorts were obtained from Zhujiang Hospital (ZJH), Southern Medical University (*n* = 123), and the Fourth Hospital of Hebei Medical University (FHHMU) (*n* = 113); for both cohorts, data were collected from January to December 2023. We additionally designed and conducted a prospective CRC cohort (*n* = 166; September 2025 to December 2025), in which eligible patients were consecutively enrolled at NFH, with no post hoc exclusions based on model output, and a retrospective multimodal gastric cancer (GC) cohort (*n* = 110; March 2023 to February 2025), also from NFH. Patients were enrolled based on predefined eligibility criteria and were not selected according to lymph node status, pathological outcomes, or model predictions. Detailed patient characteristics are provided in Table 1 and Table S1.

All WSIs used for DeepLNM model training and validation were derived exclusively from representative H&E‐stained sections of the primary CRC, rather than from lymph node sections. For each patient, the most representative primary tumor H&E slide was selected from all available primary tumor slides. The representative slide was required to show the deepest tumor cell infiltration, sufficient tumor area, and adequate staining quality. Slides with extensive necrosis, severe folding, poor staining, or insufficient tumor tissue were excluded. These slides were jointly selected by two senior gastrointestinal pathologists (W.Q. and C.Z.) according to the above criteria.

Participant sex was considered in this study. Sex information was derived from self‐report at hospital admission, recorded as part of routine demographic documentation, and summarized in the cohort characteristics.

The patients' text data were derived from admission notes written by gastrointestinal surgeons and primarily included preoperative diagnoses, such as intestinal obstruction, hypertension, and diabetes mellitus. To prevent label leakage, we explicitly excluded final pathological diagnoses, clinical staging, radiological impressions suggestive of metastasis, and any LNM‐related descriptions during preprocessing.

CT examinations were obtained as baseline preoperative imaging prior to surgical resection. Inclusion criteria were: (1) postoperative pathological confirmation of CRC or GC; (2) curative‐intent surgery for CRC or GC with adequately H&E‐stained sections; (3) availability of contrast‐enhanced or non‐contrast pelvic and abdominal CT, with imaging data retrievable in DICOM (DCM) format; (4) no receipt of neoadjuvant therapy; and (5) complete, matched clinical information (such as age, sex, and stage). Exclusion criteria were: (1) preoperative radiotherapy or neoadjuvant chemotherapy; (2) incomplete or unavailable histopathology or CT data; and (3) concurrent malignancies.

All patient‐related information obtained was ethically approved by the Institutional Ethics Committee of Nanfang Hospital, Southern Medical University (NFEC‐2024‐273 and NFEC‐2025‐403). The prospective validation study was registered at ClinicalTrials.gov (NCT07239063). Written informed consent was waived for the retrospective cohorts, whereas all participants in the prospective cohort provided written informed consent.

### 4.2 Image Acquisition and WSIs Preprocessing

Across the five datasets, we collected one representative H&E‐stained tissue section per patient, with specimen dimensions ranging from 1.0 cm × 0.5 cm × 0.5 cm to 2.0 cm × 1.5 cm × 0.5 cm. WSIs from all datasets were acquired using an Aperio AT2 scanner (Leica Biosystems; Wetzlar, Germany) at 40× magnification (corresponding to a resolution of 0.25 μm/pixel) and stored in SVS format.

For WSI processing, we followed the pipeline implemented in the Trident framework (a codebase specialized for WSI analysis). First, a low‐resolution thumbnail of the WSI was loaded into memory and converted to grayscale (or, alternatively, a specific channel‐such as the V channel‐from the HSV color space was extracted). Next, Otsu's adaptive thresholding algorithm was applied to automatically compute an optimal threshold, generating a binary foreground mask that encompasses both tumor and normal tissue regions without requiring manual specification of a fixed threshold. Subsequently, median blurring was employed to smooth edge noise, followed by morphological closing operations to fill small gaps and holes in the mask, thereby ensuring regional continuity and integrity. Finally, detected foreground contours were filtered based on an area threshold to retain only valid tissue regions, yielding the final segmentation mask for each slide. After foreground segmentation, the foreground region was partitioned into non‐overlapping patches of size 512 × 512 pixels at 20× magnification for downstream analysis.

### 4.3 Deep Learning Model Development

In this study, we propose a novel framework named DeepLNM. DeepLNM is designed to predict lymph node metastasis status by effectively integrating WSI, CT imaging data, and clinical text information. As illustrated in Figure 2, the framework follows a cascaded fusion strategy: first aligning and reconstructing visual modalities (WSI and CT), and subsequently integrating clinical semantic information.

#### 4.3.1 WSI Feature Extraction

To process gigapixel‐level whole‐slide images, we established a standardized pathological data processing pipeline [31, 36].

##### 4.3.1.1 Preprocessing and Patching

First, tissue regions are segmented at a low magnification level (Image Pyramid Layer 2) using the Otsu algorithm [37]. We apply a filtering criterion that retains only patches containing more than 25% tissue area. Subsequently, at a 20× resolution (0.25 micrometers per pixel), the valid tissue regions are cropped into overlapping patches of size 512 × 512 pixels, with an overlap of 256 pixels between adjacent patches.

##### 4.3.1.2 Normalization and Encoding

Stain normalization is performed using the structure‐preserving color normalization method of Vahadane [38]. For feature encoding, we employ the pre‐trained UNI2 backbone network to extract a high‐dimensional feature vector from each patch, leveraging recent advances in general‐purpose foundation models for pathology [21, 33].

#### 4.3.2 Radiomics feature extraction

This section describes feature extraction from CT scans and clinical reports for subsequent multimodal fusion.

##### 4.3.2.1 Deep Radiomics Encoding

The preprocessed 3D CT regions of interest are fed into the Vista3d [26] backbone network. The extracted features are projected by a multilayer perceptron into a spatial feature tensor of size W×H×3, preserving spatial structural information and aligning the CT representation in format with pathological image patches [24].

#### 4.3.3 Clinical Text Feature Extraction

For structured clinical information, we employ the CLIP text encoder [25]. Clinical records are converted into standardized textual descriptions, tokenized, and then processed by CLIP to extract semantic embeddings that provide high‐level clinical context for the visual features [20].

#### 4.3.4 Multi‐Modal Feature Fusion

To effectively integrate information from three heterogeneous modalities, we designed a two‐stage fusion module that explicitly addresses modality missingness and enables cross‐modal contextual enhancement [13, 28].

##### 4.3.4.1 Stage 1: WSI‐CT Interaction‐Based Missing Modality Aggregation

The first stage focuses on the interaction between the two imaging modalities, WSI and CT. Considering that in real‐world clinical settings, CT scans may be partially missing or of suboptimal quality, we introduce a Missing Modality Aggregator (MMA). This component employs a stochastic masking strategy during training to simulate and learn how to handle incomplete CT data. Specifically, a random binary mask is applied to the CT feature sequence, where each element is independently masked with a probability ranging from 30% to 50%. Subsequently, the unmasked WSI features serve as queries, attending to the visible portions of the CT features (used as keys and values) via an attention mechanism. This allows the model to reconstruct plausible representations of the missing radiological information based on the histopathological evidence provided by WSI. The output of this stage is an optimized and completed visual representation that jointly encodes high‐resolution histopathological details and contextually restored radiological features.

##### 4.3.4.2 Stage 2: Clinical‐Visual Cross‐Modal Fusion

In the second stage, the enhanced visual representation obtained from Stage 1 is fused with clinical text embeddings through a bidirectional cross‐attention mechanism. The clinical text here consists only of non‐diagnostic routine information, such as patient age, sex, chief complaint, and medical history. In the “visual‐to‐text” direction, visual features act as queries to compute attention over the clinical text, enabling the model to identify which clinical background elements are relevant to the current imaging or pathological findings. Conversely, in the “text‐to‐visual” direction, clinical text embeddings serve as queries to retrieve potentially relevant visual evidence from the fused WSI‐CT representation, thereby establishing explicit links between the patient's clinical context and specific morphological observations. The outputs of these two complementary attention streams are concatenated and further processed to yield a comprehensive multimodal representation that holistically integrates pathological, radiological, and basic clinical information for downstream predictive tasks.

#### 4.3.5 Feature Aggregation and Lymph Node Metastasis Prediction

To synthesize the multimodal information for the final prediction of LNM, we propose an SIM–MIL module. Unlike traditional fusion methods that simply concatenate features, SIM‐MIL treats the three distinct modalities (WSI, CT, and Clinical Text) as three individual “instances” within a patient “bag” [29]. This approach allows the model to dynamically assign importance weights to each modality based on its diagnostic contribution. We further evaluated the robustness of SIM–MIL across different mask rates, with the optimal performance achieved at a mask rate of 0.1 (Table S13).

During training, a stochastic masking strategy is applied to prevent the model from becoming overly dependent on a single dominant modality (e.g., relying solely on WSI while ignoring CT). Specifically, each modality feature is randomly retained or dropped with predefined probabilities, acting as a regularization technique that simulates scenarios where specific modality evidence might be weak or missing. During inference, all modalities are fully utilized to ensure maximum information integrity [39].

The final patient‐level representation is then obtained via attentive aggregation, in which a gated neural network computes learnable attention weights over the three modality features and produces a weighted sum as the global patient representation. This integrated vector is finally passed to the classifier to output the metastasis probability. Detailed formulations of the stochastic masking strategy and attentive aggregation are provided in the Supplementary Methods of the Supporting Information.

### 4.4 Training procedures

#### 4.4.1 Adaptive Weighted Loss Function

We propose an Adaptive Weighted Binary Cross‐Entropy (BCE) [40] Loss, in which the positive sample weight evolves dynamically during training. This design incorporates time‐decay to prioritize positive samples early in training and an uncertainty‐based correction to focus on hard examples. The detailed formulation of the adaptive weighted loss is provided in the Supplementary Methods of the Supporting Information.

#### 4.4.2 Optimization and Hyperparameters

The model was optimized using the AdamW optimizer [41] with a weight decay coefficient of 0.003 to decouple weight decay from gradient updates. We employed a Cosine Annealing learning rate scheduler [42] with a warm‐up period. The training was conducted with the following specific hyperparameters: a batch size of 16, an initial learning rate of 0.0001, and a dropout rate of 0.3 applied to the attention and fully connected layers to prevent overfitting. Furthermore, the Mish activation function [43] was adopted throughout the network to ensure better gradient flow and nonlinearity preservation.

### 4.5 Performance Evaluation

To evaluate the performance of our model, we conducted experiments on an internal dataset and multiple external validation datasets, including a prospective cohort and a specific gastric cancer cohort. For the internal dataset, we employed fivefold cross‐validation to ensure robustness and generalizability. The final performance on the internal dataset was reported as the average of the results across all five folds. For external validation, we selected the model with the best performance on the internal validation set and applied it to the external, prospective, and gastric cancer cohorts without any further tuning. This approach ensured that the external evaluation remained independent and reflected the model's real‐world generalization ability. The performance of the model was assessed using several widely adopted evaluation metrics, such as accuracy and AUC. Furthermore, to assess the clinical utility and net benefit of the model, decision curve analysis was performed specifically for the external validation, prospective, and gastric cancer cohorts.

### 4.6 User Study Design

Seven pathologists with different levels of experience participated in user study, including two senior pathologists, defined as those with more than 5 years of diagnostic experience and complete training in gastrointestinal pathology, and five junior pathologists, defined as those with 1–5 years of diagnostic experience and completion of training in CRC pathology diagnosis. All participating pathologists were blinded to the ground‐truth LNM status of the 136 cases.

The same set of slides was assessed twice by each pathologist: once independently and once with AI assistance, as shown in Figure 1f. The platform used by the pathologists in the user study is shown in Figure S6. In the first round of independent assessment, pathologists reviewed the primary tumor WSI and all available lymph node slide WSIs, and then recorded the diagnostic result, reading time, and diagnostic confidence. In the second round of AI‐assisted assessment, DeepLNM provided the pathologists with the prediction heatmap derived from the primary tumor WSI and the model‐predicted LNM status.

Notably, model predictions were still generated using the primary tumor WSI, CT, and clinical text information, without incorporating lymph node WSIs as input. The pathologists then reviewed the primary tumor WSI and all available lymph node slide WSIs, and recorded the diagnostic result, reading time, and diagnostic confidence in the same manner. A 4‐week washout period was used between the two rounds to mitigate memory bias. For each case, reading time was recorded from the moment the pathologist opened the patient's WSI data until completion of the diagnostic assessment of all lymph node slide WSIs. Diagnostic confidence scores were recorded at each assessment stage on a scale from 1 to 10, with higher scores indicating greater confidence in the diagnosis.

All statistical analysis plans were prespecified prior to result analysis. Each pathologist was treated as an independent analytical unit, and paired data from 136 cases under AI‐assisted and unassisted conditions were analyzed separately for each reader. The normality of paired differences for each outcome measure was first examined using the Shapiro–Wilk test.

For per‐pathologist diagnostic accuracy, the Wilson‐score method was used to calculate 95% confidence intervals (CIs) for accuracy under each condition (without DeepLNM and with DeepLNM assistance). For paired comparison of diagnostic accuracy between conditions, the 95% CI for paired accuracy difference was computed using the Newcombe method 10. The exact McNemar test was performed on discordant pairs. For Pathologist 7, zero discordant pairs were observed across all test cases, rendering both McNemar test and subsequent Bonferroni correction not applicable (reported as NA). Bonferroni correction was applied to adjust for multiple comparisons among the six pathologists with valid McNemar test results. Adjusted *p*‐values greater than 1 were truncated to 1.000 following standard statistical practice.

As the paired differences did not follow a normal distribution, the Wilcoxon signed‐rank test was employed to compare diagnostic time and diagnostic confidence between AI‐assisted and unassisted conditions. Given that multiple hypothesis tests were performed for the same outcome measure across 7 pathologists, Bonferroni correction was applied to control the family‐wise error rate by adjusting the *p*‐values obtained from multiple comparisons. Diagnostic time and diagnostic confidence were corrected independently.

All tests were two‐sided, and a corrected *p* < 0.05 was considered statistically significant. All statistical analyses were performed using R software (Version 4.5.2). The Shapiro–Wilk test, Wilcoxon signed‐rank test, and Bonferroni correction were implemented via the stats package (Version 4.5.2), and data visualization was conducted using the ggplot2 package (Version 4.0.2).

### 4.7 Model Interpretation Analysis

To enhance the interpretability of our model for predicting lymph node metastasis status in CRC, we employed a visualization technique to highlight critical regions within whole slide images (WSIs) that significantly influence the model's predictions. Specifically, we divided the WSIs into highly overlapping tiles (50% overlap) and calculated attention scores for each tile. These scores, ranging from 0 (low relevance) to 1 (high relevance), indicate the importance of each tile to the model's prediction. We then normalized these scores to a 0–1 scale and converted them into a heatmap using a diverging colormap. This heatmap was overlaid onto the original H&E‐stained images with a transparency of 30%, allowing for simultaneous visualization of both the attention scores and the underlying histological features. In the resulting heatmap, red areas signify regions of high attention (positive evidence) by the model, which are critical for predicting lymph node metastasis, while blue areas indicate regions of low attention (negative evidence). This approach provides a clear visual explanation of how the model makes its predictions, emphasizing both the specific regions of interest and the pathological features within those regions that are relevant to the prediction of lymph node metastasis status in CRC.

### 4.8 Computing Hardware and Software

The proposed DeepLNM framework was implemented using the Python 3.10 programming language and the PyTorch 2.1 deep learning library. All computational tasks, including model training, validation, and inference testing, were executed on a high‐performance workstation. The hardware configuration consisted of an Intel Xeon Gold 6330 Processor, 1024 GB of RAM, and four NVIDIA RTX 4090D 24 GB GPUs. Our model integrates multiple deep learning architectures and therefore requires a GPU server with a minimum of 16 GB VRAM, making it incompatible with standard laptop deployment, consistent with the computing resources detailed in Section 4.7. Depending on the size of patient slides and CT data, the processing time ranges from 15 to 25 min per case. As illustrated in Figure S6, the user study interface consists of four key modules: a medical image viewer, an information panel, an AI analysis results display, and a timer.

## Author Contributions

Li Liang and Zhengyu Zhang conceived and designed the research project and experiments. Zhengyu Zhang, Danyi Li, Jinbang Li, and Li Liang developed the methodology. Chengyu Lu implemented the software. Zhengyu Zhang, Chengyu Lu, Zhen Wang, Zhijian Cen, Yu Wang, Yueping Liu, and Li Liang performed data curation. Yangshu Zhou, Danyi Li, Qi Xie, Bingbing Li, Shujing Guo, Zhijun Gan, Er‐ran Zhu, Jinbang Li, Aihetaimu Aimaier, Rui Mao, Weihao Qiu, Chenglong Zhao, Ying Tan, Xiaolin Liu, Huiting Ma, Tiansu Geng, Peiyue Wu, and Huijun Du conducted the investigation. Zhengyu Zhang and Chengyu Lu carried out validation and formal analysis. Yu Wang, Yueping Liu, and Li Liang supervised the project. Li Liang acquired funding. Zhengyu Zhang, Chengyu Lu, and Zhijian Cen produced the visualization. Li Liang managed project administration and Jinbang Li, Yu Wang, Yueping Liu, and Li Liang contributed to resources. Zhengyu Zhang, Chengyu Lu, Zhen Wang, and Zhijian Cen wrote the original draft, while Yu Wang, Yueping Liu, and Li Liang reviewed and edited the manuscript.

## Conflicts of Interest

The authors declare no conflicts of interest.

## Supporting information


**Supporting File 1**: advs77860‐sup‐0001‐SuppMat.pptx.

**Supporting File 2**: advs77860‐sup‐0002‐SuppMat.pdf.

## Data Availability

Datasets are not publicly available due to patient privacy considerations. The source code for this study is publicly available at https://github.com/xiaoerjason/deeplnm.
