# Supplementary material for: DeepLNM: A Multimodal Model for Predicting Lymph Node Metastasis in Colorectal Cancer
Source: Adv Sci (Weinh). 2026 Oct 14:e77860. Online ahead of print. doi: 10.1002/advs.77860 (PMC13668012; doi:10.1002/advs.77860)
Supplement: Supplementary file 1 [file ADVS-9999-e77860-s002.pdf]

Table S1 Descriptive characteristics of patients with gastric cancer at NFH(n=110)

Characteristics	All(n=110)	LNM		*P* Value	SMD
		Yes (n=49)	No (n=61)		
**Gender, n (%)**				0.952	0.012
Female	67 (60.909)	30 (61.224)	37 (60.656)		
Male	43 (39.091)	19 (38.776)	24 (39.344)		
Age, Mean±SD	59.382±12.078	59.980±11.861	58.902±12.327	0.644	0.090
**Histological type, n (%)**				0.547	0.236
Adenocarcinoma NOS	59 (53.636)	26 (53.061)	33 (54.098)		
Mixed Adenocarcinoma	46 (41.818)	22 (44.898)	24 (39.344)		
Signet-ring cell carcinoma	5 (4.545)	1 (2.041)	4 (6.557)		
**Histological grade, n (%)**				0.611	0.208
G1	10 (9.091)	3 (6.122)	7 (11.475)		
G2	52 (47.273)	25 (51.020)	27 (44.262)		
G3	48 (43.636)	21 (42.857)	27 (44.262)		
**Vessel carcinoma embolus, n (%)**				**<0.001**	1.648
1	51 (46.364)	40 (81.633)	11 (18.033)		
0	59 (53.636)	9 (18.367)	50 (81.967)		
**Perineural invasion, n (%)**				**<0.001**	0.737
1	53 (48.182)	33 (67.347)	20 (32.787)		
0	57 (51.818)	16 (32.653)	41 (67.213)		
**pT, n (%)**				**<0.001**	1.209
T1	41 (37.273)	6 (12.245)	35 (57.377)		
T2	21 (19.091)	9 (18.367)	12 (19.672)		
T3	25 (22.727)	19 (38.776)	6 (9.836)		
T4	23 (20.909)	15 (30.612)	8 (13.115)		
**Stage, n (%)**				**<0.001**	0.635
Stage I	43 (39.091)	0 (0.000)	43 (70.492)		
Stage II	18 (16.364)	0 (0.000)	18 (29.508)		
Stage III	49 (44.545)	49 (100.000)	0 (0.000)		
**CT, n (%)**				0.703	0.073
Contrast-enhanced CT	95 (86.364)	43 (87.755)	52 (85.246)		
Non-contrast CT	15 (13.636)	6 (12.245)	9 (14.754)		

NFH: Nanfang Hospital, Southern Medical University; LNM: Lymph Node Metastasis; SMD: Standardized Mean Difference;
NOS: Not Otherwise Specified

Table S2 Results of different base models on internal datasets

Method	Sensitivity (95% CI)	Specificity (95% CI)	Accuracy (95% CI)	AUC (95% CI)
UNI	0.904 (0.878-0.922)	0.806 (0.781-0.831)	0.845 (0.824-0.869)	0.894 (0.872-0.917)
CONCH	0.887 (0.864-0.910)	0.794 (0.768-0.820)	0.833 (0.809-0.856)	0.882 (0.858-0.906)
Virchow2	0.881 (0.857-0.905)	0.788 (0.761-0.815)	0.826 (0.803-0.850)	0.876 (0.852-0.901)
Prov-Gigapath	0.921 (0.901-0.941)	0.828 (0.804-0.852)	0.867 (0.845-0.887)	0.915 (0.896-0.935)
H-Optimus	0.878 (0.853-0.903)	0.784 (0.757-0.811)	0.822 (0.799-0.847)	0.873 (0.849-0.897)
**UNI2-h**	**0.928 (0.909-0.947)**	**0.835 (0.802-0.858)**	**0.871 (0.853-0.894)**	**0.922 (0.905-0.940)**

To compare the performance of different pathology foundation models in our study, we conducted a performance analysis across multiple models. The results showed that UNI2-h achieved the best performance; therefore, we selected this foundation model for subsequent experimental analyses.

Table S3 Detailed results of the external cohort (ZJH)

Model	Sensitivity (95% CI)	Specificity (95% CI)	Accuracy (95% CI)	AUC (95% CI)	*P* value
ABMIL	0.712 (0.589–0.835)	0.776 (0.679–0.873)	0.747 (0.665–0.829)	0.698 (0.622–0.774)	<0.001
AttentionMIL	0.662 (0.533–0.791)	0.702 (0.596–0.808)	0.684 (0.598–0.770)	0.698 (0.600–0.796)	<0.001
CLAM_MB	0.598 (0.465–0.731)	0.856 (0.774–0.938)	0.748 (0.663–0.833)	0.667 (0.590–0.744)	<0.001
CLAM_SB	0.676 (0.549–0.803)	0.746 (0.634–0.833)	0.701 (0.626–0.777)	0.685 (0.603–0.766)	<0.001
DSMIL	0.658 (0.529–0.787)	0.732 (0.619–0.821)	0.690 (0.621–0.759)	0.679 (0.591–0.768)	<0.001
Maxpool	0.688 (0.562–0.814)	0.718 (0.605–0.810)	0.694 (0.619–0.769)	0.697 (0.626–0.769)	<0.001
TransMIL	0.728 (0.607–0.849)	0.775 (0.665–0.856)	0.752 (0.730–0.794)	0.786 (0.717–0.835)	0.002
**DeepLNM**	**0.813 (0.707–0.919)**	**0.796 (0.702–0.890)**	**0.803 (0.744–0.863)**	**0.872 (0.819–0.918)**	**-**

ZJH: Zhujiang Hospital, Southern Medical University

Table S4 Detailed results of the external cohort (FHHMU)

Model	Sensitivity (95% CI)	Specificity (95% CI)	Accuracy (95% CI)	AUC (95% CI)	*P* value
ABMIL	0.712 (0.648–0.768)	0.684 (0.618–0.742)	0.698 (0.634–0.744)	0.774 (0.726–0.817)	0.055
AttentionMIL	0.765 (0.718–0.832)	0.729 (0.674–0.798)	0.747 (0.706–0.825)	0.728 (0.647–0.779)	0.003
CLAM_MB	0.654 (0.588–0.716)	0.702 (0.638–0.758)	0.678 (0.617–0.736)	0.674 (0.619–0.744)	<0.001
CLAM_SB	0.698 (0.632–0.756)	0.650 (0.584–0.714)	0.674 (0.616–0.745)	0.688 (0.638–0.754)	<0.001
DSMIL	0.742 (0.688–0.788)	0.696 (0.642–0.748)	0.719 (0.672–0.762)	0.656 (0.569–0.744)	<0.001
Maxpool	0.718 (0.648–0.776)	0.764 (0.702–0.818)	0.741 (0.674–0.798)	0.690 (0.644–0.754)	<0.001
TransMIL	0.688 (0.618–0.748)	0.638 (0.568–0.704)	0.663 (0.597–0.716)	0.768 (0.702–0.836)	0.045
**DeepLNM**	**0.836 (0.762–0.898)**	**0.806 (0.728–0.872)**	**0.821 (0.748–0.894)**	**0.848 (0.755–0.928)**	**-**

FHHMU: Fourth Hospital of Hebei Medical University

Table S5 Ablation experiment results

ID	Configuration	Sensitivity (95% CI)	Specificity (95% CI)	AUC (95% CI)
M1	Text Baseline (Single Modality)	0.584 (0.473–0.688)	0.670 (0.576–0.752)	0.691 (0.611–0.770)
M2	WSI Baseline (Single Modality)	0.812 (0.785-0.842)	0.854 (0.821-0.887)	0.895 (0.872-0.918)
M3	CT Baseline (Single Modality)	0.725 (0.692-0.758)	0.784 (0.750-0.818)	0.835 (0.802-0.858)
M4	Simple Fusion (Concat Only)	0.845 (0.810-0.875)	0.876 (0.842-0.905)	0.902 (0.885-0.920)
M5	Cross-Attention (Basic Fusion)	0.872 (0.844-0.898)	0.898 (0.865-0.924)	0.915 (0.896-0.932)
M6	M4 + Random Masking	0.868 (0.838-0.895)	0.902 (0.872-0.928)	0.912 (0.890-0.930)
M7	M5 + SIM-MIL	0.885 (0.858-0.911)	0.914 (0.886-0.938)	0.922 (0.905-0.940)
**M8**	**Full Model (SIM-MIL + Contrastive)**	**0.892 (0.865-0.919)**	**0.918 (0.895-0.941)**	**0.928 (0.909-0.947)**

Ablation experiment results of DeepLNM under different configurations. In the internal cohort, M1 (Text baseline) was tested using complete text modality data, while M2 (WSI baseline) outperformed M3 (CT baseline). By progressively adding components, the model performance increased from M4 (Concat only), to M5 (Cross-Attention), to M6 (M4 + Random Masking), and then to M7 (M5 + SIM-MIL). The full model, M8 (SIM-MIL + Contrastive), achieved the best overall performance, supporting the contribution of each module and the benefit of complementary multimodal information.

Table S6 Detailed results of the prospective cohort

Model	Sensitivity (95% CI)	Specificity (95% CI)	Accuracy (95% CI)	AUC (95% CI)	*P* value
ABMIL	0.735 (0.648–0.810)	0.708 (0.615–0.788)	0.721 (0.633–0.803)	0.796 (0.730–0.862)	0.096
AttentionMIL	0.742 (0.655–0.818)	0.685 (0.592–0.768)	0.712 (0.626–0.790)	0.803 (0.742–0.901)	0.233
CLAM_MB	0.718 (0.645–0.785)	0.752 (0.680–0.815)	0.735 (0.669–0.800)	0.771 (0.699–0.852)	0.027
CLAM_SB	0.705 (0.630–0.775)	0.740 (0.665–0.808)	0.723 (0.654–0.801)	0.768 (0.697–0.843)	0.018
DSMIL	0.738 (0.655–0.810)	0.710 (0.620–0.785)	0.724 (0.640–0.790)	0.791 (0.707–0.853)	0.096
Maxpool	0.685 (0.615–0.750)	0.712 (0.640–0.778)	0.698 (0.633–0.760)	0.787 (0.688–0.879)	0.170
TransMIL	0.745 (0.670–0.815)	0.695 (0.620–0.770)	0.719 (0.656–0.800)	0.815 (0.722–0.866)	0.349
**DeepLNM**	**0.772 (0.725–0.815)**	**0.745 (0.695–0.792)**	**0.759 (0.710–0.807)**	**0.845 (0.801–0.888)**	**-**

Table S7 Performance comparison between single-WSI and multi-WSIs settings

Dataset	Sensitivity (95% CI)	Specificity (95% CI)	Accuracy (95% CI)	AUC (95% CI)
Single-WSI	0.772 (0.725–0.815)	0.745 (0.695–0.792)	0.759 (0.710–0.807)	0.845 (0.801–0.888)
Multi-WSIs	0.786 (0.695–0.877)	0.771 (0.679–0.863)	0.762 (0.669–0.855)	0.812 (0.723–0.901)

The table presents a comparative evaluation of single-patient single-slice (Single-WSI) versus single-patient multi-slice (Multi-WSIs) settings within a prospective cohort. At a 95% confidence level, no statistically significant difference was observed between Single-WSI and Multi-WSIs ($P > 0.05$), demonstrating that our DeepLNM method is equally effective under both data modalities.

Table S8 Detailed results of the gastric cancer cohort

Model	Sensitivity (95% CI)	Specificity (95% CI)	Accuracy (95% CI)	AUC (95% CI)	*P* value
ABMIL	0.735 (0.642–0.828)	0.709 (0.618–0.800)	0.722 (0.636–0.808)	0.702 (0.587–0.811)	0.100
AttentionMIL	0.708 (0.615–0.801)	0.722 (0.631–0.813)	0.715 (0.630–0.799)	0.709 (0.594–0.819)	0.130
CLAM_MB	0.749 (0.651–0.833)	0.721 (0.627–0.809)	0.730 (0.643–0.817)	0.714 (0.600–0.823)	0.152
CLAM_SB	0.695 (0.604–0.786)	0.719 (0.628–0.811)	0.707 (0.623–0.791)	0.717 (0.605–0.825)	0.165
DSMIL	0.728 (0.637–0.819)	0.708 (0.617–0.799)	0.718 (0.636–0.801)	0.698 (0.581–0.811)	0.091
Maxpool	0.685 (0.594–0.776)	0.699 (0.608–0.790)	0.692 (0.609–0.774)	0.656 (0.541–0.764)	0.013
TransMIL	0.738 (0.647–0.829)	0.714 (0.623–0.805)	0.726 (0.640–0.812)	0.726 (0.619–0.831)	0.212
**DeepLNM**	**0.782 (0.691–0.873)**	**0.754 (0.663–0.845)**	**0.768 (0.677–0.860)**	**0.793 (0.683–0.892)**	**-**

Table S9 Descriptive baseline characteristics of patients in the user study from NFH (n=136)

Characteristics	All(n=136)	LNM		*P* Value	SMD
		Yes (n=55)	No (n=81)		
**Age, Mean±SD**	61.125±11.976	59.545±12.414	62.198±11.625	0.206	0.222
**Gender, n (%)**				0.201	0.224
Female	53 (38.971)	25 (45.455)	28 (34.568)		
Male	83 (61.029)	30 (54.545)	53 (65.432)		
**Histological type, n (%)**				0.647	0.249
Adenocarcinoma NOS	134 (98.529)	54 (98.182)	80 (98.765)		
Signet-ring Cell Carcinoma	1 (0.735)	1 (1.818)	0 (0.000)		
Other	1 (0.735)	0 (0.000)	1 (1.235)		
**Histological grade, n (%)**				0.814	0.178
G2	129 (94.853)	52 (94.545)	77 (95.062)		
G3	6 (4.412)	3 (5.455)	3 (3.704)		
G4	1 (0.735)	0 (0.000)	1 (1.235)		
**Vessel carcinoma embolus, n (%)**				<0.001	0.785
YES	29 (21.324)	22 (40.000)	7 (8.642)		
NO	107 (78.676)	33 (60.000)	74 (91.358)		
**Perineural invasion, n (%)**				0.001	0.58
YES	69 (50.735)	37 (67.273)	32 (39.506)		
NO	67 (49.265)	18 (32.727)	49 (60.494)		
**pT, n (%)**				0.07	0.561
TIS	2 (1.471)	0 (0.000)	2 (2.469)		
T1	4 (2.941)	0 (0.000)	4 (4.938)		
T2	19 (13.971)	4 (7.273)	15 (18.519)		
T3	81 (59.559)	38 (69.091)	43 (53.086)		
T4	30 (22.059)	13 (23.636)	17 (20.988)		
**Stage, n (%)**				<0.001	0.777
Stage I	21 (15.441)	0 (0.000)	21 (25.926)		
Stage II	60 (44.118)	0 (0.000)	60 (74.074)		
Stage III	55 (40.441)	55 (100.000)	0 (0.000)		

NFH: Nanfang Hospital, Southern Medical University; LNM: Lymph Node Metastasis; SMD: Standardized Mean Difference; NOS: Not Otherwise Specified

Table S10 Summary of user study data from NFH

LNM, n(%)	Number of cases (n=136)	Number of WSIs (n=426)
**Yes**	55(40.441)	188(44.131)
**No**	81(59.559)	238(55.869)

NFH: Nanfang Hospital, Southern Medical University; LNM: Lymph Node Metastasis

Table S11 Per-pathologist diagnostic accuracy with and without AI assistance in the user study

Pathologist	Condition	Correct/Total	Accuracy,% (95% Wilson CI)	Accuracy difference,% (Newcombe 95% CI)	McNemar *P*	Bonferroni *P*
P1	Without AI	125/136	91.91 (86.10-95.42)	+5.88 (-0.11-11.26)	0.008	0.048
	With AI	133/136	97.79 (93.72-99.25)			
P2	Without AI	129/136	94.85 (89.76-97.48)	+1.47 (-4.04-6.81)	0.500	1.000
	With AI	131/136	96.32 (91.68-98.42)			
P3	Without AI	126/136	92.65 (86.99-95.96)	+5.15 (-0.69-10.40)	0.039	0.234
	With AI	133/136	97.79 (93.72-99.25)			
P4	Without AI	126/136	92.65 (86.99-95.96)	+3.68 (-2.35-9.38)	0.062	0.372
	With AI	131/136	96.32 (91.68-98.42)			
P5	Without AI	132/136	97.06 (92.68-98.85)	+1.47 (-3.03-5.61)	0.500	1.000
	With AI	134/136	98.53 (94.80-99.60)			
P6	Without AI	133/136	97.79 (93.72-99.25)	+0.74 (-3.48-4.74)	1.000	1.000
	With AI	134/136	98.53 (94.80-99.60)			
P7	Without AI	136/136	100.00 (97.25-100.00)	+0.00 (-2.75-2.75)	NA	NA
	With AI	136/136	100.00 (97.25-100.00)			

NA: Not Available, McNemar-derived and Bonferroni-adjusted P-values could not be calculated because there were zero discordant pairs.

Table S12 Comparison of histological features stratified by AI-predicted patch metastasis labels

Variables	Total (*n*=600)	Metastasis		Test statistic	*P* value	SMD
		Positive (*n*=300)	Negative (*n*=300)			
**Histological features, *n* (%)**				χ^2=238.326[1]	<0.001	1.624
normal neuromuscular tissue	85 (14.167%)	18 (6.000%)	67 (22.333%)			
poorly-differentiated glandular structures	145 (24.167%)	130 (43.333%)	15 (5.000%)			
necrosis	67 (11.167%)	49 (16.333%)	18 (6.000%)			
adipocyte infiltration	45 (7.500%)	5 (1.667%)	40 (13.333%)			
desmoplastic reaction	92 (15.333%)	64 (21.333%)	28 (9.333%)			
lymphocytic infiltration	90 (15.000%)	11 (3.667%)	79 (26.333%)			
well-differentiated glandular structures	76 (12.667%)	23 (7.667%)	53 (17.667%)			

SMD: Standardized Mean Difference

Table S13 SIM-MIL framework mask rate ablation experiment

ID	Configuration	Sensitivity (95% CI)	Specificity (95% CI)	AUROC (95% CI)
1	0.01	0.863 (0.836-0.890)	0.889 (0.866-0.912)	0.901 (0.882-0.920)
2	**0.1**	**0.892 (0.865-0.919)**	**0.918 (0.895-0.941)**	**0.928 (0.909-0.947)**
3	0.2	0.881 (0.854-0.908)	0.906 (0.883-0.929)	0.915 (0.896-0.934)
4	0.3	0.834 (0.807-0.861)	0.858 (0.835-0.881)	0.873 (0.854-0.892)
5	0.4	0.778 (0.770-0.786)	0.801 (0.778-0.821)	0.816 (0.797-0.835)
6	0.5	0.798 (0.771-0.825)	0.822 (0.799-0.845)	0.838 (0.819-0.857)
7	0.8	0.771 (0.770-0.772)	0.793 (0.770-0.816)	0.808 (0.789-0.827)

The table illustrates the performance of the SIM-MIL framework under varying mask rates, with the optimal performance achieved at a mask rate of 0.1. SIM-MIL: Stochastic Instance Masking Multiple Instance Learning

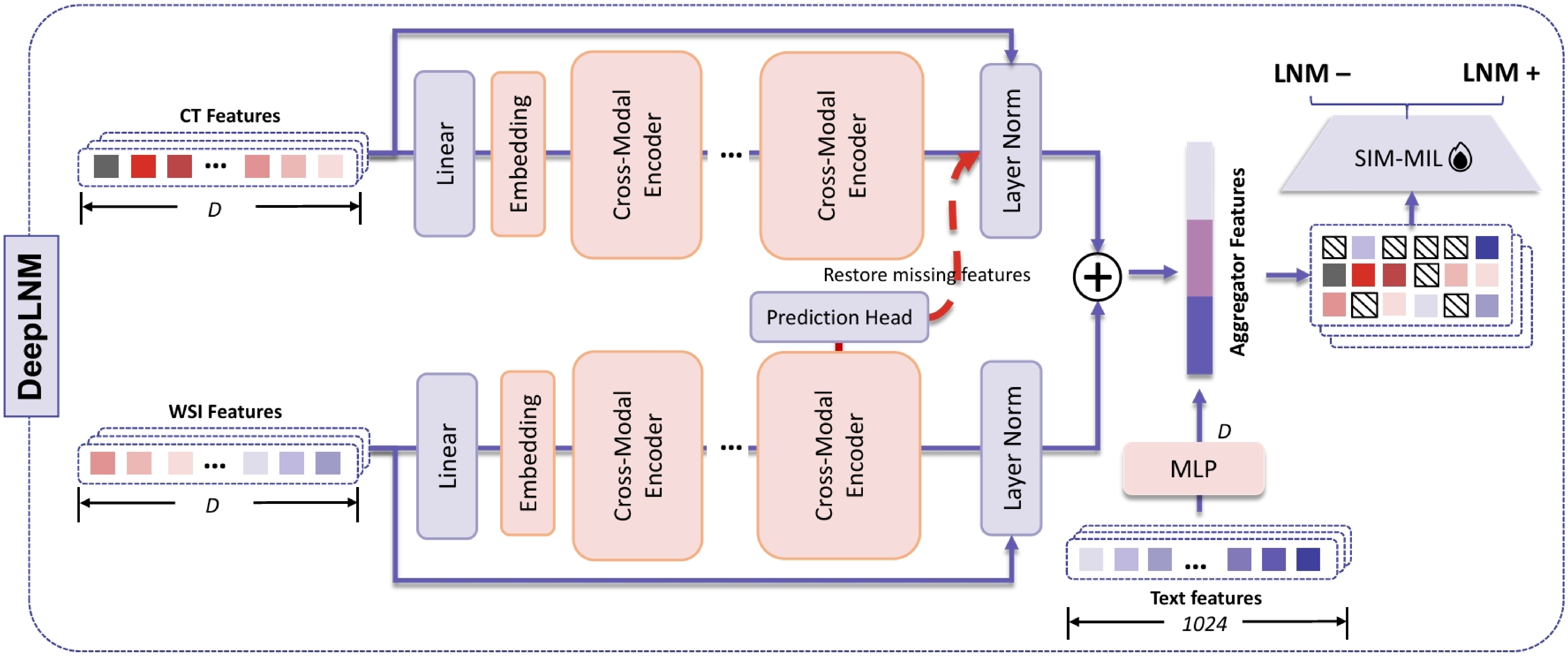

Figure S1. Schematic illustration of the DeepLNM architecture.

The framework employs a multi-modal fusion strategy integrating CT imaging features, WSI pathological features, and clinical text features. The CT and WSI features are processed through parallel streams comprising linear projection, embedding layers, and stacked Cross-Modal Encoders to capture inter-modal interactions. Specifically, a feature restoration mechanism equipped with a Prediction Head is incorporated into the WSI branch to effectively mitigate the issue of missing modality data. The visual representations, normalized via Layer Norm, are fused through element-wise addition and subsequently aggregated with clinical text features encoded by a Multi-Layer Perceptron. Finally, the aggregated masked instance features are input into the Scale-Invariant Multi-Instance Learning (SIM-MIL) module to predict Lymph Node Metastasis (LNM) status (LNM- vs. LNM+).

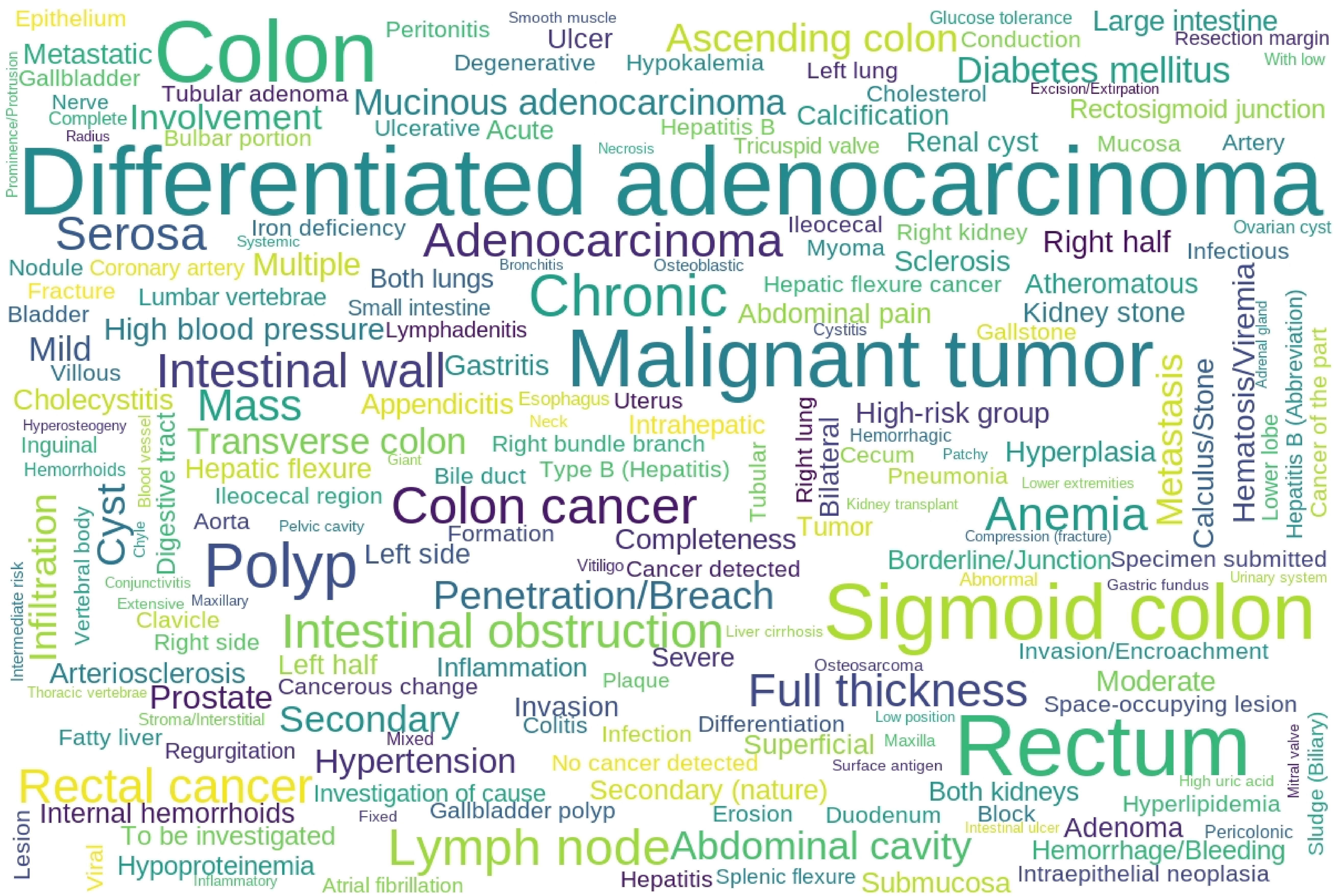

Figure S2. Word cloud visualization of clinical diagnoses and pathological features. The word cloud represents the frequency of clinical and pathological terms identified in the study population (frequency 3). The font size is proportional to the occurrence frequency of each term. The visualization highlights differentiated colorectal adenocarcinoma as the primary diagnosis. Major pathological indicators emphasize tumoral infiltration (e.g., "full thickness", "serosa") and associated complications (e.g., "intestinal obstruction", "anemia"), while peripheral terms reflect prevalent comorbidities such as hypertension and diabetes mellitus.

a

b

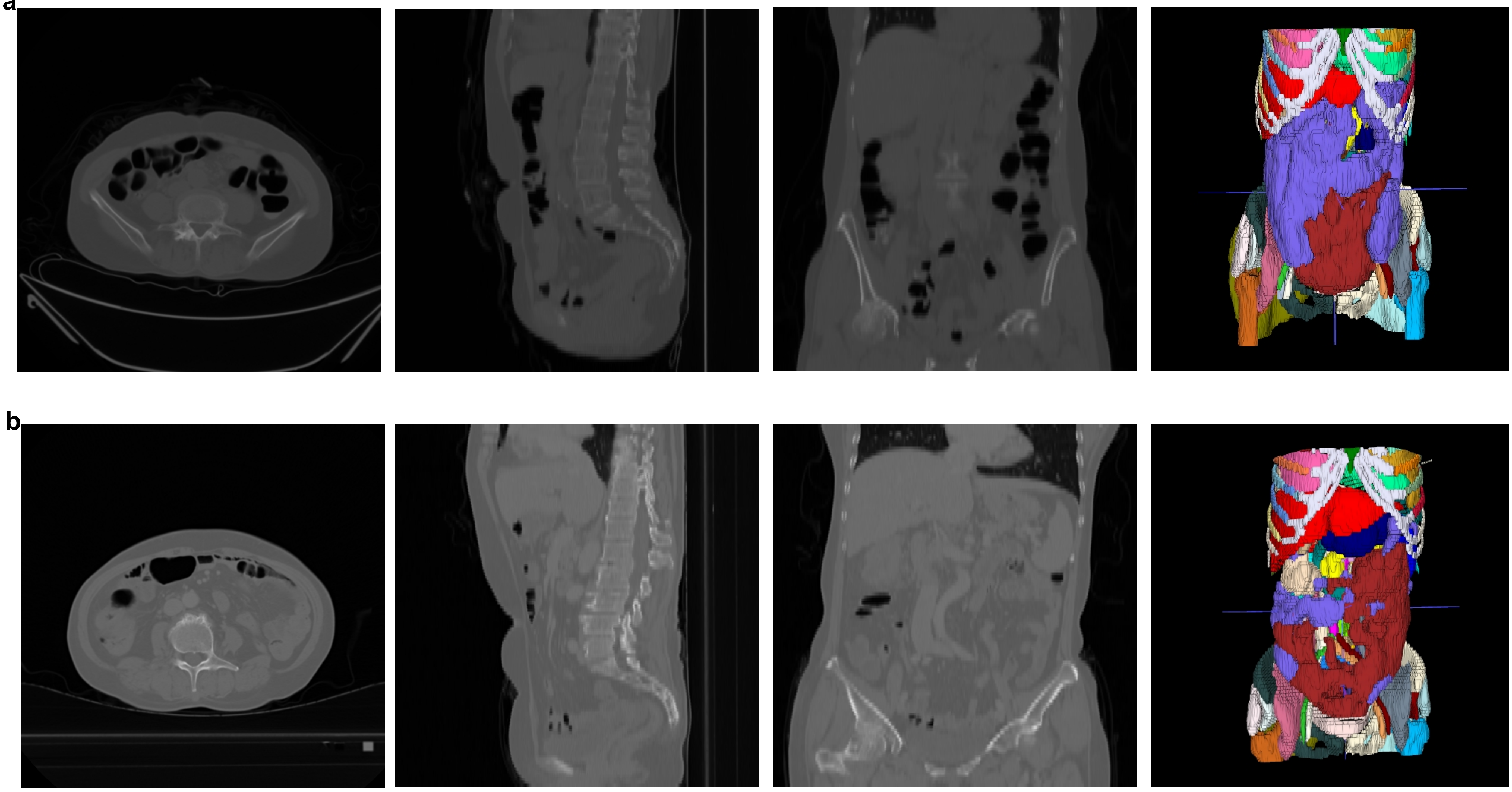

Figure S3. CT-based anatomical segmentation and feature visualization using Vista3D. Panels (a) and (b) present representative cases showcasing the multi-planar CT reconstructions and their corresponding 3D visualizations. Left three columns: Axial, sagittal, and coronal CT views, respectively, providing multi-angle anatomical context. Rightmost column: 3D feature visualization and organ/tissue segmentation generated by the Vista3D framework. Different colors represent distinct anatomical structures (e.g., skeletal system, abdominal organs, and vascular structures), highlighting the model's capability in precise spatial feature extraction and volumetric rendering.

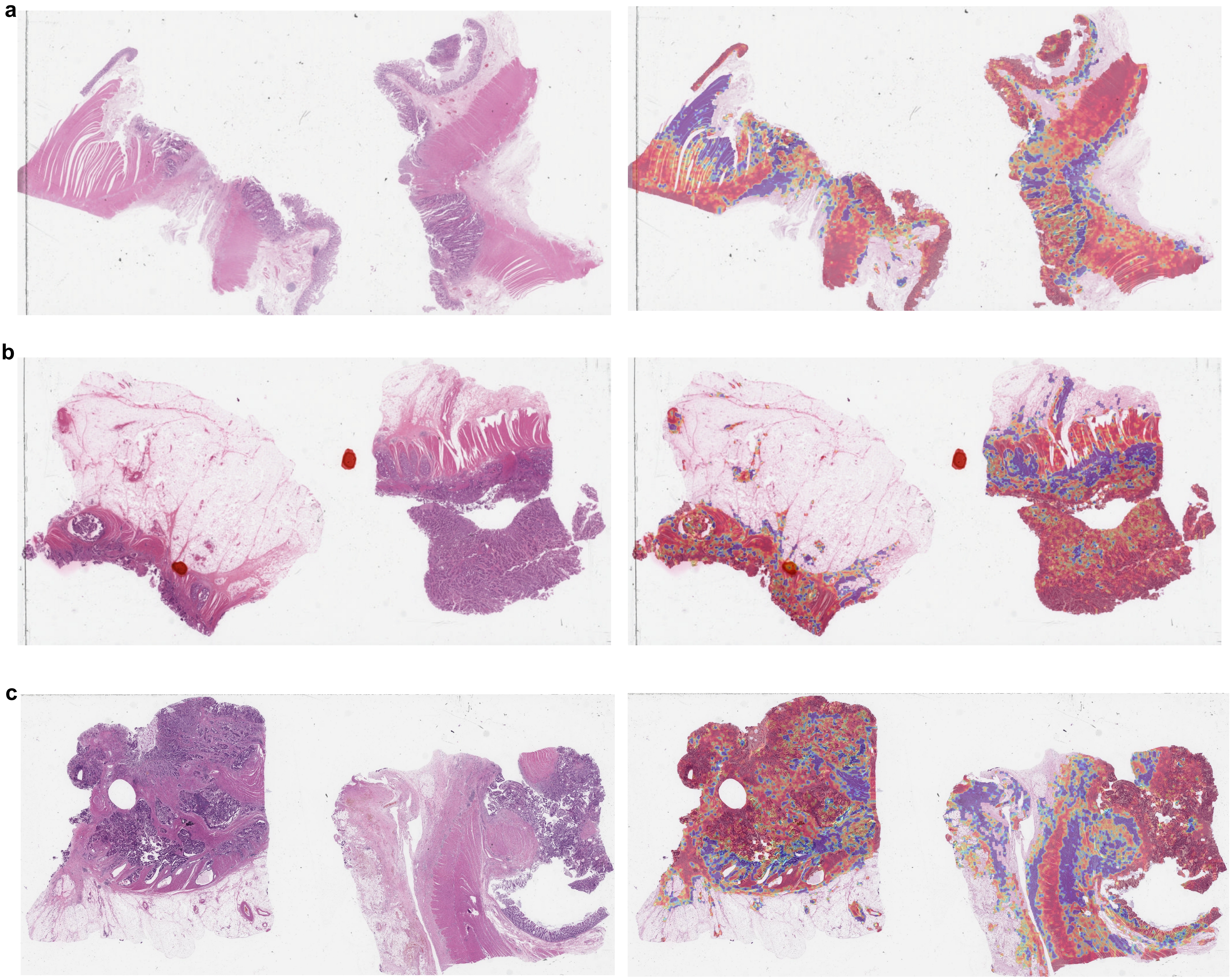

Figure S4. DeepLNM-based heatmap generation for WSI analysis. Original input WSIs (left) and DeepLNM-derived inference heatmaps (right) are presented. (a, b) Representative WSIs negative for LNM; (c) WSI positive for LNM.

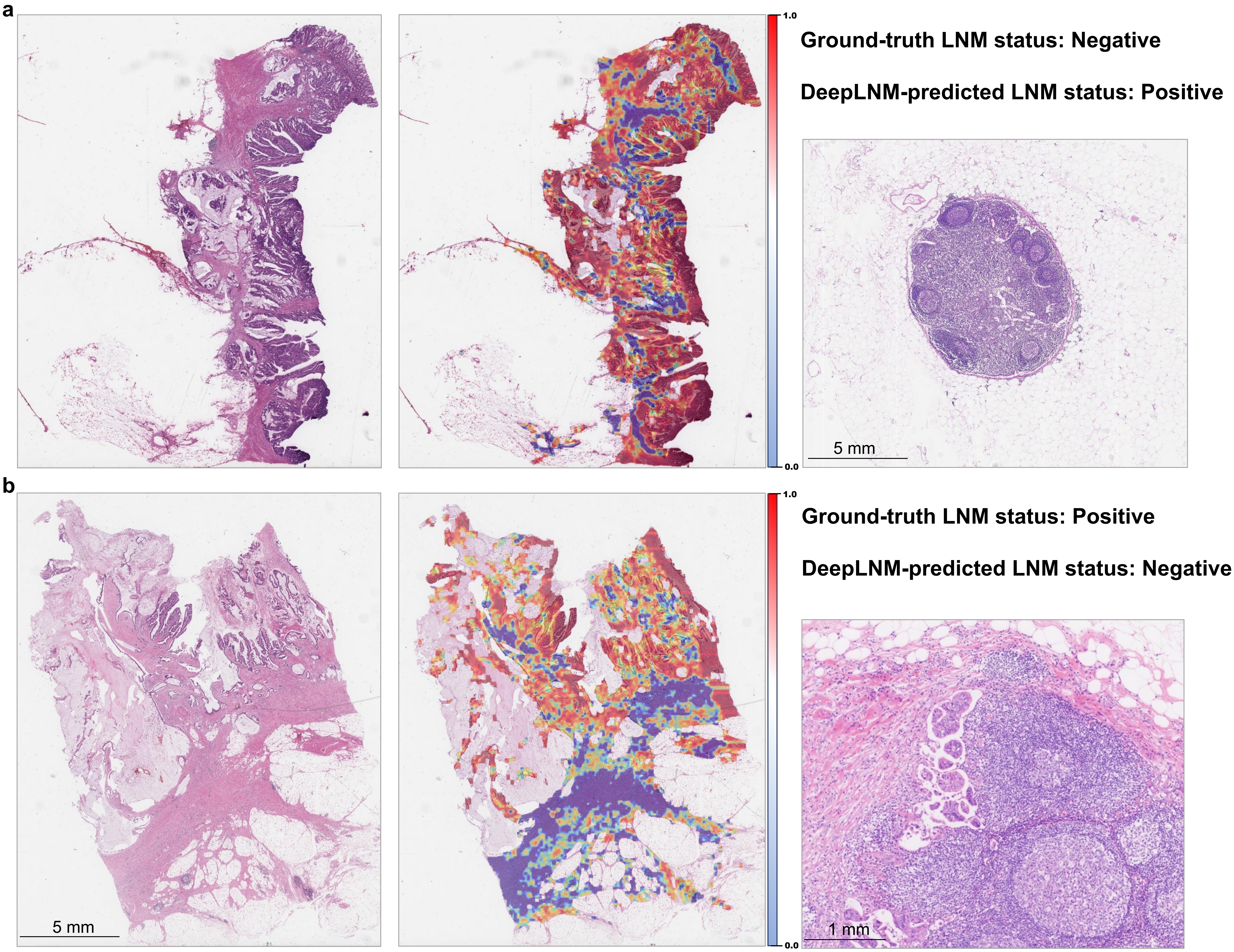

Figure S5. Representative examples of incorrect predictions by DeepLNM. (a) A false-positive case predicted as LNM positive by DeepLNM. From left to right: the primary tumor WSI, the corresponding DeepLNM prediction heatmap, and a representative lymph node WSI showing no tumor cells. (b) A false-negative case predicted as LNM negative by DeepLNM. From left to right: the primary tumor WSI, the corresponding DeepLNM prediction heatmap, and a representative lymph node WSI showing colorectal cancer tumor cells.

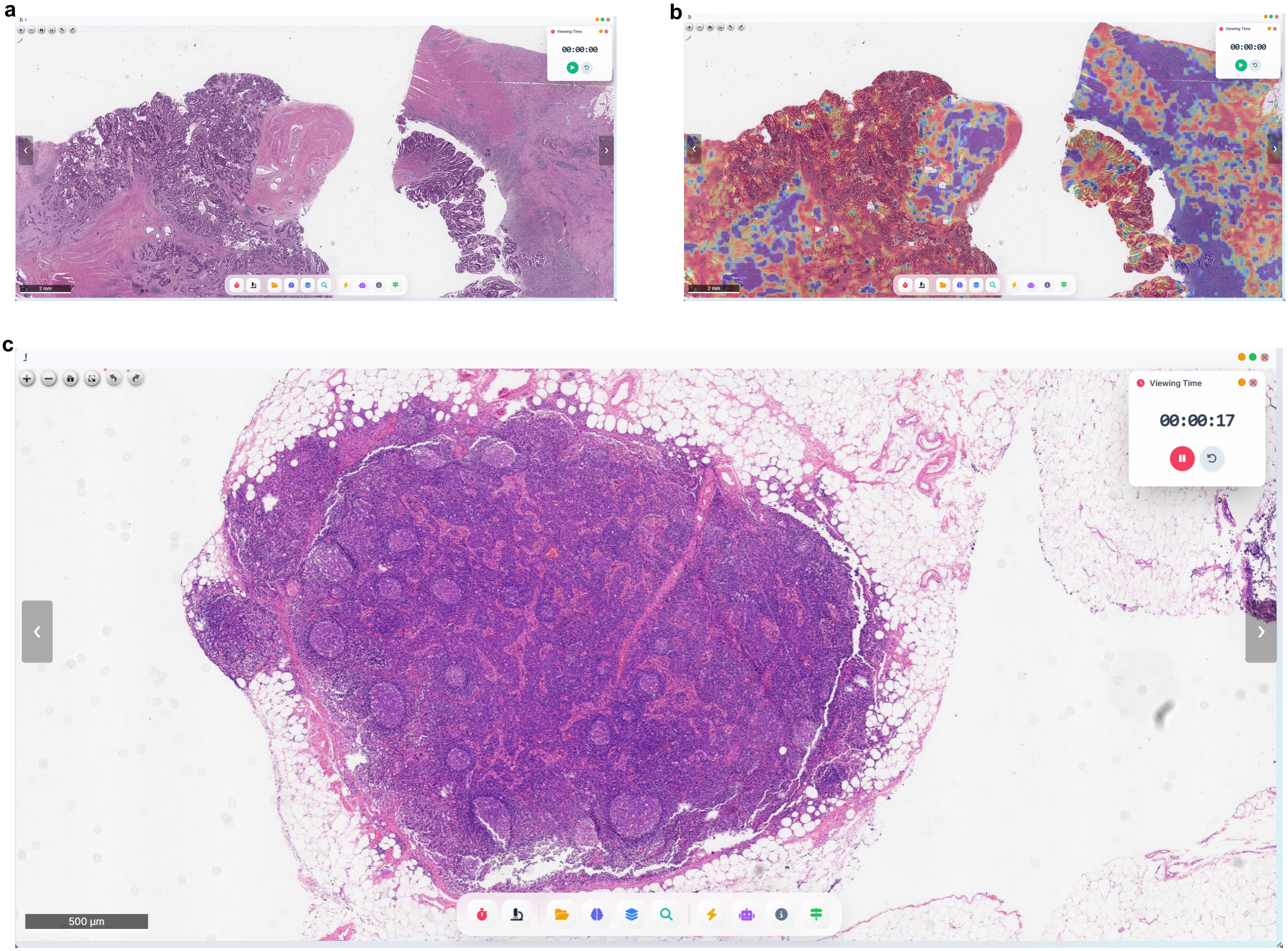

Figure S6. Schematic of the AI-assisted digital pathology platform with integrated diagnostic timer. The interface illustrates the AI-driven clinical workflow: (a) represents the original histopathological image of tumor tissue; (b) shows the overlay result of the DeepLNM-derived inference heatmap used to highlight suspicious regions; and (c) displays a lymph node pathology image featuring an integrated "Viewing Time" module. This timer assists pathologists in quantifying diagnostic duration and evaluating overall workflow efficiency.
